# Comparison of HIV Screening Strategies in the Emergency Department: A Randomized Clinical Trial

**DOI:** 10.1001/jamanetworkopen.2021.17763

**Published:** 2021-07-26

**Authors:** Jason S. Haukoos, Michael S. Lyons, Richard E. Rothman, Douglas A. E. White, Emily Hopkins, Meggan Bucossi, Andrew H. Ruffner, Rachel M. Ancona, Yu-Hsiang Hsieh, Stephen C. Peterson, Danielle Signer, Matthew F. Toerper, Mustapha Saheed, Sarah K. Pfeil, Tamara Todorovic, Alia A. Al-Tayyib, Lucy Bradley-Springer, Jonathan D. Campbell, Edward M. Gardner, Sarah E. Rowan, Allison L. Sabel, Mark W. Thrun

**Affiliations:** 1 Department of Emergency Medicine, Denver Health Medical Center, Denver, Colorado; 2 Department of Emergency Medicine, University of Colorado School of Medicine, Aurora; 3 Department of Epidemiology, Colorado School of Public Health, Aurora; 4 Department of Emergency Medicine, University of Cincinnati College of Medicine, Cincinnati, Ohio; 5 Department of Emergency Medicine, Johns Hopkins University, Baltimore, Maryland; 6 Department of Emergency Medicine, Highland Hospital, Oakland, California; 7 Denver Public Health, Denver, Colorado; 8 Division of Infectious Diseases, University of Colorado School of Medicine, Aurora; 9 Department of Clinical Pharmacy, Skaggs School of Pharmacy and Pharmaceutical Sciences, Aurora, Colorado; 10 Department of Patient Safety and Quality, Denver Health, Denver, Colorado; 11 Department of Biostatistics, Colorado School of Public Health, Aurora; 12 Gilead Sciences, Inc, Foster City, California

## Abstract

**Importance:**

The National HIV Strategic Plan for the US recommends HIV screening in emergency departments (EDs). The most effective approach to ED-based HIV screening remains unknown.

**Objective:**

To compare strategies for HIV screening when integrated into usual ED practice.

**Design, Setting, and Participants:**

This randomized clinical trial included patients visiting EDs at 4 US urban hospitals between April 2014 and January 2016. Patients included were ages 16 years or older, not critically ill or mentally altered, not known to have an HIV positive status, and with an anticipated length of stay 30 minutes or longer. Data were analyzed through March 2021.

**Interventions:**

Consecutive patients underwent concealed randomization to either nontargeted screening, enhanced targeted screening using a quantitative HIV risk prediction tool, or traditional targeted screening as adapted from the Centers for Disease Control and Prevention. Screening was integrated into clinical practice using opt-out consent and fourth-generation antigen-antibody assays.

**Main Outcomes and Measures:**

New HIV diagnoses using intention-to-treat analysis, absolute differences, and risk ratios (RRs).

**Results:**

A total of 76 561 patient visits were randomized; median (interquartile range) age was 40 (28-54) years, 34 807 patients (51.2%) were women, and 26 776 (39.4%) were Black, 22 131 (32.6%) non-Hispanic White, and 14 542 (21.4%) Hispanic. A total of 25 469 were randomized to nontargeted screening; 25 453, enhanced targeted screening; and 25 639, traditional targeted screening. Of the nontargeted group, 6744 participants (26.5%) completed testing and 10 (0.15%) were newly diagnosed; of the enhanced targeted group, 13 883 participants (54.5%) met risk criteria, 4488 (32.3%) completed testing, and 7 (0.16%) were newly diagnosed; and of the traditional targeted group, 7099 participants (27.7%) met risk criteria, 3173 (44.7%) completed testing, and 7 (0.22%) were newly diagnosed. When compared with nontargeted screening, targeted strategies were not associated with a higher rate of new diagnoses (enhanced targeted and traditional targeted combined: difference, −0.01%; 95% CI, −0.04% to 0.02%; RR, 0.7; 95% CI, 0.30 to 1.56; *P* = .38; and enhanced targeted only: difference, −0.01%; 95% CI, −0.04% to 0.02%; RR, 0.70; 95% CI, 0.27 to 1.84; *P* = .47).

**Conclusions and Relevance:**

Targeted HIV screening was not superior to nontargeted HIV screening in the ED. Nontargeted screening resulted in significantly more tests performed, although all strategies identified relatively low numbers of new HIV diagnoses.

**Trial Registration:**

ClinicalTrials.gov Identifier: NCT01781949

## Key Points

**Question:**

What is the most effective HIV screening strategy for emergency departments (EDs)?

**Findings:**

This randomized clinical trial included 76 561 ED patient visits allocated to either nontargeted HIV screening or 1 of 2 forms of targeted HIV screening. A total of 14 405 HIV tests were completed resulting in 24 (0.17%) individuals identified with newly diagnosed HIV, with no difference in rates of diagnosis among the 3 strategies.

**Meaning:**

Targeted and nontargeted HIV screening yielded comparable numbers of new HIV diagnoses in the ED.

## Teaser

This randomized clinical trial examines 76 561 emergency department patient visits to compare effectiveness of targeted and nontargeted HIV screening strategies for finding new diagnoses.

## Introduction

Diagnosis of HIV is an important health priority and critical step in the care continuum.^1,2^ While increased screening efforts in the US have reduced the number of those with undiagnosed HIV, a significant number of persons living with HIV remain undiagnosed.^3^

The Centers for Disease Control and Prevention (CDC) and US Preventive Services Task Force (USPSTF) recommend routine nontargeted HIV screening in most medical settings, including emergency departments (EDs).^4,5,6,7^ The alternative, targeted (risk-based) HIV screening,^8^ was historically endorsed by the CDC and USPSTF.^9,10^ Effectiveness of targeted HIV screening in ED practice has gone largely unexplored and comparative studies of varied implementation approaches, including targeted vs nontargeted screening, are limited.^11,12,13^ Recently, the Denver HIV Risk Score (DHRS), a quantitative risk prediction tool, was validated to estimate HIV risk, and was shown in a nonrandomized ED study to be strongly associated with new HIV diagnoses when used to perform targeted screening and when compared with nontargeted screening.^14,15^

The goal of this study was to compare targeted and nontargeted HIV screening strategies when integrated into practice across multiple EDs, with the hypothesis that targeted screening would identify more persons with previously undiagnosed HIV than nontargeted screening.

## Methods

### Study Design

The HIV Testing using Enhanced Screening Techniques in Emergency Departments (TESTED) trial was a multicenter, prospective, pragmatic (ie, the interventions were evaluated in real-life routine practice conditions), 3-arm randomized clinical trial (study protocol in Supplement 1). This study is reported in accordance with the Consolidated Standards of Reporting Trials (CONSORT) reporting guideline and the PRECIS-2 framework.^16,17^ Each site’s institutional review board approved the study with a waiver of informed consent because of the pragmatic nature of the trial.

### Setting

We performed this study in 4 high-volume EDs in Baltimore, Maryland (Johns Hopkins Hospital); Cincinnati, Ohio (University of Cincinnati Medical Center); Denver, Colorado (Denver Health Medical Center); and Oakland, California (Highland Hospital) with a combined annual ED census of over 300 000 visits. These sites serve large, heterogeneous, and underserved populations (ie, populations that face barriers to accessing health care) with varying proportions of racial/ethnic minority patients and HIV epidemiologies.

### Population

Patients were eligible for randomization if clinically stable and able to consent for medical care. Patients were excluded if they (1) were younger than 16 years of age; (2) were unable to provide consent for care or HIV testing (eg, altered mental activity, critical illness, or injury); (3) were known to be living with HIV; (4) were the victim of sexual assault; (5) had occupational exposure to HIV; or (6) had an anticipated ED length of stay under 30 minutes.

### Interventions

#### Randomization

Randomization occurred from April 8, 2014, through January 27, 2016, and was incorporated into the electronic health record (EHR) system at each institution using a computer-generated random number algorithm developed and validated at each site prior to initiating enrollment. Real-time randomization occurred 24 hours per day, providing concealed allocation with equal probability assignment to 1 of the 3 HIV screening arms. This process triggered various EHR prompts during the triage nurse workflow.

#### Nontargeted HIV Screening

Patients allocated to this arm were notified, without assessment of risk, that HIV testing would be performed unless they declined (ie, opted out). To minimize bias and to preserve the pragmatic approach, nurses were instructed not to ascertain risk for patients included in this arm.

#### Enhanced Targeted HIV Screening

Patients allocated to this arm underwent risk-based HIV screening using the empirically derived and externally validated DHRS, which includes 6 variables: age, gender, race/ethnicity, sexual orientation, injection drug use, and prior HIV testing (eTable 1 in Supplement 2).^15^ The composite DHRS, which reflects a patient’s HIV risk, was automatically calculated by the EHR and a score of 30 or above triggered an EHR prompt for the triage nurse to notify the patient that HIV testing would be performed unless declined; those who scored below 30 were considered low risk and not tested for HIV.

#### Traditional Targeted HIV Screening

Patients allocated to this arm underwent risk-based HIV screening using conventional risk questions (eTable 2 in Supplement 2) adapted from and recommended by the CDC prior to 2006 and the USPSTF prior to 2013.^9,18^ A tool was developed, referred to as the Behavioral Risk Screening Tool (BRST), and an affirmative response to any risk question was considered positive and triggered an EHR prompt for the triage nurse to notify the patient that HIV testing would be performed unless declined; patients who did not indicate any risk were not tested for HIV.

#### HIV Testing

All HIV screening was voluntary and confidential with consent obtained in a verbal manner and separate from general consent for care, and all processes were fully integrated into usual emergency care with HIV test results returning during the ED visit. Central laboratory-based fourth-generation antigen-antibody assays were used at all institutions (Denver Health: Alere North America, LLC; Johns Hopkins Hospital, University of Cincinnati Medical Center, and Highland Hospital: Abbott Laboratories). Standard, institution-specific, linkage-to-care processes were performed for all patients who tested preliminarily positive for HIV. Confirmatory testing was initiated during the ED visit and completed during linkage to care.^19^

### Outcomes

The primary outcome was confirmed new HIV diagnoses. In anticipation of testing patients with previously diagnosed HIV (ie, repeat diagnoses), we included a composite secondary outcome of new and repeat diagnoses (ie, all diagnoses). Diagnoses were classified as acute infection (ie, antigen positive but antibody negative), established infection (ie, antibody positive), or AIDS (ie, antibody positive with a CD4 cell count under 200 cells/μL). Additional outcomes included behavioral risk among those randomized to the targeted arms and those identified with HIV, CD4 cell counts and viral loads (copies/mL) at the time of diagnosis, and successful linkage into care (defined as completion of an initial HIV clinic visit). Follow-up for a period of 1 year after diagnosis was assessed and included initiation of antiretroviral therapy, unscheduled medical care visits and hospitalizations, and mortality.

### Data Collection

Data were collected from each institution’s EHR and prevention program records, and included: patient visit information (ie, unique identifier, acuity level, mode of ED arrival, and date/time of visit); demographics (age, sex, race/ethnicity as classified by patients as part of their ED care [because HIV disproportionately affects individuals from specific racial/ethnic groups], primary language); payer information (commercial, Medicare, Medicaid, self, or state-sponsored); details of randomization, including the intervention assigned and the results of risk screening, if applicable; whether the patient declined testing, if offered; completion of testing; results of all HIV tests; and, for patients with a reactive HIV test, results of confirmatory tests, initial CD4 cell counts and viral loads, whether they were successfully linked into care, additional behavioral risk information, and details of follow-up care and disease progression through January 17, 2017 (ie, 1 year after randomization concluded).

### Statistical Analysis

Data were transferred or manually entered into an electronic database or spreadsheet, respectively, (Microsoft SQL or Excel, Microsoft Corporation) and further transferred to the data coordinating center (Denver, CO) where concatenation and analyses were performed using SAS Enterprise Guide version 7.1 (SAS Institute, Inc). Analyses were performed using the intention-to-treat principle and with no planned interim analysis. Aggregate patient-level data are reported for demographics; otherwise, patient visit data are reported as the primary unit of analysis. Analyses were completed on April 1, 2021.

Comparing targeted HIV screening with nontargeted HIV screening and, more specifically, enhanced targeted HIV screening with nontargeted HIV screening, primary comparisons included absolute differences and multilevel log-binomial regression to estimate risk ratios (RRs) for new HIV diagnoses while accounting for patients clustered within site. Given the randomized design, no covariates were included in the models to account for confounding.

Secondary comparisons included differences and RRs for targeted and enhanced targeted HIV screening for new and repeat HIV diagnoses combined, as well as targeted and enhanced targeted HIV screening for new and repeat HIV diagnoses combined using only those who were notified that testing would be performed as the denominator. Additionally, we compared results for patients identified as high risk between the 2 targeted arms who did not decline testing and completed testing. Comparisons included stage of illness at the time of diagnosis, linkage to care, initiation and adherence with HIV medications, unscheduled health care visits, and mortality at 12-month follow-up across all arms. A χ^2^ or Fisher exact test for categorical data were used to compare data between groups and 95% CIs were reported for all estimates.

### Sample Size

Using simulation, pilot data, and past experience with each screening method,^12,13,20,21^ we estimated a minimum of 12 600 HIV tests to reach 80% power identifying a significant effect of targeted HIV screening (RR, 1.33; 95% CI, 1.01 to 1.79; α = .05). We adjusted the sample size using a design effect to account for within-institution correlation and increased the sample size by an additional 10% to account for limitations in our assumptions, resulting in a requirement of 14 000 HIV tests. The trial was planned with balanced enrollment, thus requiring 3500 tests from each site.

## Results

**Figure 1.**
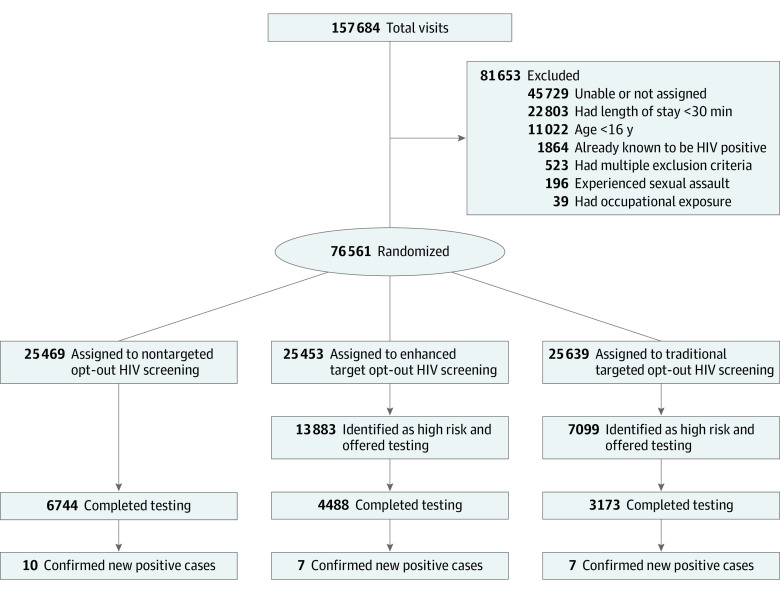
CONSORT Flow Diagram Enrollment occurred from April 8, 2014, through January 27, 2016, with longitudinal follow-up through January 27, 2017. Patients considered high risk for enhanced targeted screening had a Denver HIV Risk Score of 30 or higher; for traditional targeted screening, eligible patients had at least 1 risk behavior as adapted from the Centers for Disease Control and Prevention.

A total of 76 561 visits by 67 964 unique individuals met criteria for inclusion and were randomized (Figure 1). The median (interquartile range [IQR]) age was 40 (28-54) years; 34 807 patients (51.2%) were women, and 26 776 (39.4%) were Black, 22 131 (32.6%) non-Hispanic White, and 14 542 (21.4%) Hispanic (Table 1). Most individuals had Medicaid (31 201 [45.9%]), followed by commercial insurance (14 665 [21.6%]) and self-pay (7917 [11.6%]); 15 880 (20.7%) arrived by ambulance and most patients were categorized as having an Emergency Severity Index (ESI) level 3 (47 912 [62.6%]), with excellent balance between study arms. Comparisons of patients and visits not eligible or eligible but not randomized are shown in eTable 3 in Supplement 2.

**Table 1.** Baseline Characteristics of Randomized Patients by Study Arm

Characteristics	No. (%)^a^		
	Nontargeted	Enhanced targeted	Traditional targeted
**Patient-level characteristics (Nontargeted, 22 658; Enhanced targeted, 22 616; Traditional targeted, 22 690)**			
Age, median (IQR), y	40 (28-54)	40 (28-54)	40 (28-54)
Sex			
Women	11 619 (51.3)	11 540 (51.0)	11 648 (51.3)
Men	11 039 (48.7)	11 076 (49.0)	11 040 (48.7)
Unknown/missing	0	0	2 (<0.1)
Race/ethnicity			
Asian, non-Hispanic	586 (2.6)	602 (2.7)	592 (2.6)
Black, non-Hispanic	8909 (39.3)	8800 (38.9)	9067 (40.0)
Hispanic (all races)	4891 (21.6)	4863 (21.5)	4788 (21.1)
White, non-Hispanic	7342 (32.4)	7434 (32.9)	7355 (32.4)
Other^b^	486 (2.1)	466 (2.1)	480 (2.1)
Unknown/missing	444 (2.0)	451 (2.0)	408 (1.8)
Payer			
Commercial	4865 (21.5)	4820 (21.3)	4980 (21.9)
Medicaid	10 370 (45.8)	10 437 (46.1)	10 394 (45.8)
Medicare	2915 (12.9)	2861 (12.7)	2848 (12.6)
Self-pay	2652 (11.7)	2645 (11.7)	2620 (11.5)
Other	719 (3.2)	673 (3.0)	723 (3.2)
Unknown/missing	1137 (5.0)	1180 (5.2)	1125 (5.0)
**Visit-level characteristics (Nontargeted, 25 469 visits; Enhanced targeted, 25 453; Traditional targeted, 25 639)**			
Mode of arrival			
Ambulatory	19 116 (75.1)	19 222 (75.5)	19 360 (75.5)
EMS	5319 (20.9)	5263 (20.7)	5298 (20.7)
Unknown/missing	1034 (4.1)	968 (3.8)	981 (3.8)
Acuity			
ESI Level 1	232 (0.9)	235 (0.9)	230 (0.9)
ESI Level 2	3154 (12.4)	3146 (12.4)	3242 (12.6)
ESI Level 3	15 984 (62.8)	15 912 (62.5)	16 016 (62.5)
ESI Level 4	5540 (21.8)	5589 (22.0)	5594 (21.8)
ESI Level 5	536 (2.1)	546 (2.1)	527 (2.1)
Unknown/missing	23 (<0.1)	25 (<0.1)	30 (0.1)

Abbreviations: EMS, emergency medical services; ESI, Emergency Severity Index; IQR, interquartile range.

^a^ Demographics are reported at the patient level (n = 67 964) and emergency department characteristics at the visit level (n = 76 561).

^b^ Other was defined as American Indian/Alaskan Native, Native Hawaiian/Pacific Islander, or multiple race/ethnicities.

### HIV Screening

**Figure 2.**
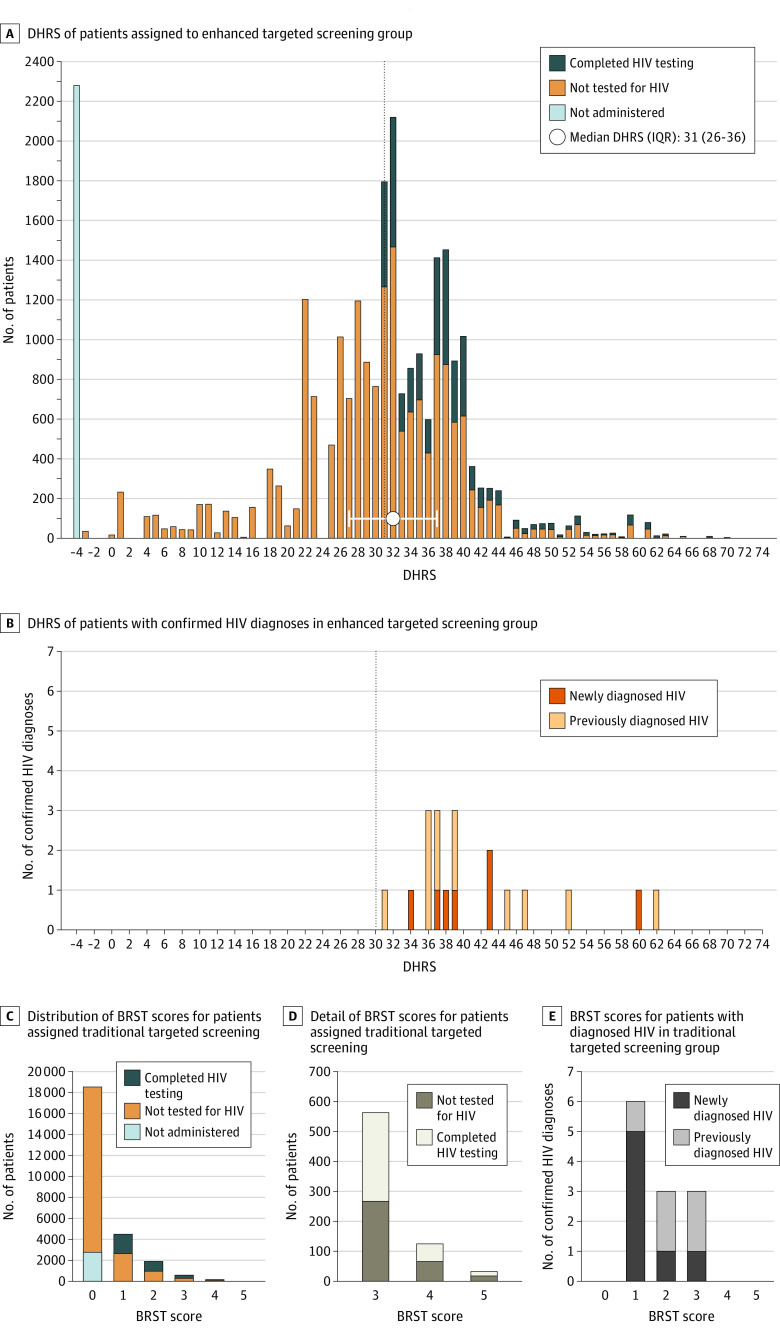
Distribution of Patient Visits and HIV Diagnoses Within Targeted Screening Arms BRST indicates Behavioral Risk Screening Tool; DHRS, Denver HIV risk score.

Of the 76 561 visits, 14 405 included completed testing, with 55 (0.38%) confirmed reactive tests, of which 24 (0.17%) were new diagnoses (Table 2). Of the 25 469 patients allocated to nontargeted screening, 9313 (36.6%) did not decline testing and 6744 (26.5%) completed testing, of whom 10 (0.15%; 95% CI, 0.07% to 0.27%) were identified with newly diagnosed HIV. Of the 25 453 patients allocated to enhanced targeted screening, 13 883 (54.5%) were identified as having increased risk for HIV, of whom 6010 (43.3%) did not decline testing and 4488 (32.3%) completed testing, with a total of 7 patients (0.16%; 95% CI, 0.06% to 0.32%) identified with newly diagnosed HIV. Of the 25 639 patients allocated to traditional targeted screening, 7099 (27.7%) were identified as having increased risk for HIV, of whom 4164 (58.7%) did not decline testing and 3173 (44.7%) completed testing, with a total of 7 patients (0.22%; 95% CI, 0.08% to 0.45%) identified with newly diagnosed HIV. Figure 2 shows distributions of patient visits and HIV diagnoses within the enhanced and traditional targeted screening arms and eTable 4 in Supplement 2 shows baseline characteristics for high-risk and low-risk targeted patients.

**Table 2.** Number and Prevalence of All Confirmed and New HIV Diagnoses in Each Arm by Those Randomized, Those Offered HIV Testing, and Those Who Completed HIV Testing

	Nontargeted		Enhanced targeted		Traditional targeted		
	No.	% (95% CI)	No.	% (95% CI)	No.		% (95% CI)
**Randomized visits (Nontargeted, 25 469; Enhanced targeted, 25 453; Traditional targeted, 25 639)**							
All confirmed HIV diagnoses, No.	24	0.09 (0.06-0.14)	19	0.07 (0.04-0.12)	12		0.05 (10.02-0.08)
New HIV diagnoses, No.	10	0.03 (0.02-0.07)	7	0.03 (0.01-0.06)	7		0.03 (0.01-0.06)
**Opt-out HIV screening visits**^**a**^** (Nontargeted, 25 469; Enhanced targeted, 13 883; Traditional targeted, 7099)**							
All confirmed HIV diagnoses, No.	24	0.09 (0.06-0.14)	19	0.14 (0.08-0.21)	12		0.17 (0.09-0.30)
New HIV diagnoses, No.	10	0.03 (0.02-0.07)	7	0.05 (0.02-0.10)	7		0.10 (0.04-0.20)
**Completed HIV testing visits (Nontargeted, 6744; Enhanced targeted, 4488; Traditional targeted, 3173)**							
All confirmed HIV diagnoses, No.	24	0.36 (0.22-0.53)	19	0.42 (0.26-0.67)	12		0.38 (0.19-0.65)
New HIV diagnoses, No.	10	0.15 (0.07-0.27)	7	0.16 (0.06-0.32)	7	0.22 (0.09-0.45)	

^a^ Excludes patients who were identified as low risk by either enhanced targeted or traditional targeted screening, thus representing only those patients who could have been tested for HIV.

#### Targeted vs Nontargeted Screening

When compared with nontargeted screening, targeted screening resulted in a larger percentage who did not decline testing (10 174 [49.0%] vs 9313 [37.0%] patients; difference, 12.0%; 95% CI, 11.1% to 12.9%; *P* < .001), as well as a larger percentage of patients who completed testing (7661 [36.5%] vs 6744 [26.5%]; difference, 10.0%; 95% CI, 9.2% to 10.9%; *P* < .001). However, of all patients randomized to the targeted arms, the percentage of HIV tests performed was smaller (7661 [15.0%] vs 6744 [26.5%]; difference, 11.5%; 95% CI, 10.9% to 12.1%; *P* < .001) when compared with nontargeted screening (eTable 5 in Supplement 2).

#### Enhanced vs Traditional Targeted Screening

Enhanced targeted screening identified about twice as many patients at increased risk when compared with traditional targeted screening (13 883 [54.5%] vs 7099 [27.7%]; difference, 26.8%; 95% CI, 26.0% to 27.7%; *P* < .001), although of those identified at increased risk, a larger percentage in the traditional targeted arm did not decline testing (4164 [58.7%] vs 6010 [43.3%]; difference, 15.4%; 95% CI, 14.0% to 16.8%; *P* < .001). Overall, between the 2 targeted approaches, more HIV tests were performed using enhanced targeting (4488 tests, 17.6% of all visits) when compared with traditional targeting (3173 tests, 12.4% of all visits) (difference, 5.3%; 95% CI, 4.6% to 5.9%; *P* < .001).

#### HIV Diagnoses: Targeted vs Nontargeted Screening

When compared with nontargeted screening, targeted strategies were not significantly associated with new HIV diagnoses (enhanced and traditional targeted screening combined: difference, −0.01%; 95% CI, −0.04% to 0.02%; RR, 0.70; 95% CI, 0.30 to 1.56; *P* = .38; enhanced targeted only: difference, −0.01%; 95% CI, −0.04% to 0.02; RR, 0.70; 95% CI, 0.27 to 1.84; *P* = .47). Furthermore, targeted strategies, either combined or individually, were not significantly associated with any confirmed HIV diagnoses, and no significant associations were identified after restricting to only those who were notified that HIV testing would be performed (Table 3). eFigures 1 through 4 in Supplement 2 show results stratified by institution.

**Table 3.** Relative Risks for Targeted vs Nontargeted HIV Screening by Those Randomized and Those Offered HIV Testing^a^

Comparisons	RR (95% CI)	
	New HIV Diagnoses	All HIV Diagnoses
Randomized (n = 76 561)		
Targeted vs Nontargeted	0.70 (0.30-1.56)	0.65 (0.38-1.10)
Enhanced targeted vs Nontargeted	0.70 (0.27-1.84)	0.79 (0.43-1.45)
Enhanced targeted vs Traditional targeted	1.01 (0.35-2.87)	1.59 (0.77-3.29)
Opt-out HIV screening^b^ (n = 46 451)		
Targeted vs Nontargeted	1.70 (0.76-3.83)	1.57 (0.92-2.67)
Enhanced targeted vs Nontargeted	1.28 (0.49-3.37)	1.45 (0.80-2.65)
Enhanced targeted vs Traditional targeted	0.51 (0.18-1.46)	0.81 (0.39-1.67)

Abbreviation: RR, relative risk.

^a^ Analyses were performed using a multilevel log-binomial regression model with site as a random effect to account for patients clustered within sites. The intraclass correlation coefficient was 0.

^b^ Excludes patients who were identified as low risk by either enhanced targeted or traditional targeted screening, thus representing only those patients who could have been tested for HIV.

### Disease Stage, Linkage to Care, and Long-Term Follow-up

Of the 24 new diagnoses, the median (IQR) initial CD4 cell count and viral load were 339 (197-529) cells/μL (to convert to cells × 10^9^/L, multiply by 0.001) and 28 959 (5359-106 000) copies/mL, respectively, with 1 case identified as acute (4.2%; 95% CI, 1.1% to 21.1%) and 4 (16.7%; 95% CI, 4.7% to 37.4%) as AIDS. Of the 24 new diagnoses, 22 (91.7%; 95% CI, 73.0% to 99.0%) were successfully linked into care, 15 (62.5%; 95% CI, 40.6% to 81.2%) initiated treatment, and 4 (16.7%; 95% CI, 4.7% to 37.4%) died during follow-up. Additional linkage-to-care, treatment, and follow-up details are shown in eTables 6 and 7 in Supplement 2.

## Discussion

This study represents the largest HIV screening trial to date with the overarching goal of understanding the comparative effectiveness of 2 forms of targeted HIV screening—a traditional targeted approach endorsed by the CDC prior to 2006 and a novel approach using a validated quantitative HIV risk score—and nontargeted HIV screening. Targeted and nontargeted HIV screening resulted in clinically comparable numbers of new HIV diagnoses, although significantly fewer tests were required as part of the targeted strategies, making targeted screening potentially more efficient. Notably, all 3 screening strategies exceeded the 0.1% HIV test prevalence threshold for performing routine screening as suggested by the CDC and supported by cost analyses.^4,22,23^ Furthermore, relatively small proportions of newly diagnosed patients were AIDS-defined at the time of diagnosis, suggesting the benefits of screening to prevent late diagnosis, and the majority of patients had improved CD4 cell counts and viral loads 1 year after diagnosis.

Rationale for the head-to-head comparisons performed in this trial resulted from concerns that nontargeted screening may be too broad for ED settings for several reasons: (1) the number of individuals with undiagnosed HIV has significantly declined over the past decade^8^ and declining numbers of undiagnosed HIV may have decreased the efficiency of nontargeted screening over time^3^; (2) the likely need for targeted screening approaches for repeat testing; (3) a preliminary nonrandomized trial supported targeted screening over nontargeted screening^13^; and (4) a paucity of research has compared targeted with nontargeted screening.^12^

 Several nonrandomized studies have evaluated various approaches to ED-based HIV screening, including opt-out vs opt-in consent,^20,24^ point-of-care testing vs laboratory testing,^25,26^ and use of supplemental staff or kiosks to perform screening.^24,25,27,28,29^ However, to our knowledge, only 2 trials have previously evaluated targeted screening in the ED, with limitations related to generalizability and comparisons.^11,12^ Furthermore, several studies have evaluated nontargeted HIV screening in ED settings, and results from the nontargeted arm of our trial are similar in terms of acceptance or not declining (approximately 35%), test completion (approximately 25%), and new HIV diagnoses (approximately 0.2%).^20,24,25,26,27,28,29,30,31,32,33,34,35,36,37^ Additionally, nontargeted screening results in more patients screened for HIV overall, although a large proportion (approximately 75%) were previously tested for HIV, and in a prior study a large number of patients tested as part of nontargeted screening were considered low risk.^13^

Despite not identifying a clearly superior approach, this trial does support the importance of routine screening for HIV in EDs. Although the overall number of new HIV diagnoses was low, the individuals who were diagnosed through screening efforts were able to start antiretroviral therapy and improve their health and, very likely, to avert transmissions. These actions would not have been possible without an HIV diagnosis. Additional understanding of how screening interventions factor into ED operational efficiency,^38^ cost effectiveness,^21,39^ and acceptance by both patients and ED staff^40,41,42^ will be needed to inform decisions about which approach is ideal for this clinical setting.

No HIV screening approach is likely to be implemented with perfect fidelity. This calls attention to the benefits of complimentary approaches within the same practice setting. Concurrent with this study’s nurse-driven screening, physician-directed testing identified a significant number of cases and a much higher percentage of positive tests. Physician-directed diagnostic testing in EDs has historically led to low volumes of testing. We do not know the motivation of physicians when ordering these tests but presume that most, if not all, were for the purposes of diagnostic testing based on signs or symptoms of HIV or that targeted behavioral risk. The presence of undetected HIV in the ED population despite the presence of large screening efforts raises questions about the interactions between efficacy and patient selection criteria under ideal circumstances, the effectiveness of patient selection criteria under real-world circumstances, and completeness of implementation. As progress toward ending the HIV epidemic in the US has stalled over the past few years, it is now especially critical to utilize all diagnostic approaches and venues, leveraging EDs in ways that are pragmatic and sustainable to decrease undiagnosed HIV.

### Limitations

This study has several limitations. Although this trial randomized a large heterogeneous population across geographically different locations, it was conducted at 4 academic medical centers with experience performing HIV screening. Thus, although generalizable, the results may not be directly applicable to community EDs. Of the 157 684 total visits, 51.4% were excluded, largely due to illness severity, altered mental activity, or an estimated short length of stay. Inclusion was driven, in part, by nursing judgement, although it is possible they inadvertently or intentionally excluded patients who could have been included. Performance of point-of-care testing in the ED, which was done in part at 1 site, may have mitigated exclusions. However, given the high volume of screening and the large numbers of tests performed, the primary testing approach was to rely on each hospital’s central laboratory to complete HIV testing because it was most consistent with a sustainable HIV screening program. Furthermore, the decision to offer HIV testing to certain patients randomized to specific arms may have been influenced by perceived or real risks identified through the medical screening examination process. However, we believe the potential for this form of contamination was minimized by inclusion of hundreds of nurses across the multiple sites. Finally, the number of new HIV diagnoses was smaller than anticipated and may have limited the ability to identify significant differences.

## Conclusions

Targeted HIV screening was not superior to nontargeted HIV screening in the ED. Nontargeted screening resulted in significantly more tests performed, although all strategies identified relatively low numbers of new HIV diagnoses.

## Notes

Supplement 1. Trial Protocol and Analytic Plan

Supplement 2. **eTable 1.** Denver HIV Risk Score **eTable 2.** Traditional Targeted HIV Screening Questions **eTable 3.** Baseline Characteristics of Ineligible, Eligible but Not Randomized, and Randomized Patients **eTable 4.** Baseline Characteristics of Patients Randomized to Enhanced Targeted and Traditional Targeted Arms, Stratified by Risk **eTable 5.** Test Offer and Completion Among Patients Randomized to Each Study Arm, All Sites **eTable 6.** Outcomes During 12 Months Following HIV Diagnosis, Among All New HIV Diagnosed Patients by Study Arms, All Sites **eTable 7.** Outcomes During 12 Months Following HIV Diagnosis, Among All Patients Who Underwent Diagnostic Testing, All Sites **eFigure 1.** Enrollment From Denver Health Medical Center **eFigure 2.** Enrollment From the University of Cincinnati Medical Center **eFigure 3.** Enrollment From Johns Hopkins Hospital, Baltimore, Maryland **eFigure 4.** Enrollment From Highland Hospital, Oakland, California

Supplement 3. **Nonauthor Collaborators.** The HIV TESTED (Testing using Enhanced Screening Techniques in Emergency Departments) Trial Investigators

Supplement 4. Data Sharing Statement
