# Supplementary material for: Comparison of HIV Screening Strategies in the Emergency Department: A Randomized Clinical Trial
Source: JAMA Netw Open. 2021 Jul 26;4(7):e2117763. doi: 10.1001/jamanetworkopen.2021.17763 (PMC8314142; doi:10.1001/jamanetworkopen.2021.17763)
Supplement: Supplement 1. — Trial Protocol and Analytic Plan [file jamanetwopen-e2117763-s001.pdf]

Confidential – Do Not Distribute

The HIV TESTING using ENHANCED SCREENING TECHNIQUES in EMERGENCY DEPARTMENTS (TESTED) Trial

The HIV TESTED Trial

STUDY PROTOCOL

Version 2.5

This study protocol, and all information contained within it, is CONFIDENTIAL. Please do not circulate or reproduce without prior permission from Jason Haukoos, MD, MSc

NIH Project Title: Effectiveness of Rapid HIV Screening Methods in Urban Emergency Departments
Grant Number: R01AI106057
ClinicalTrials.gov Number: NCT01781949

Principal Investigator:

Jason Haukoos, MD, MSc

Telephone: (303) 602-5174
Facsimile: (303) 602-5184
Email: Jason.Haukoos@dhha.org

Project Managers:

Emily Caruso, MSPH

Telephone: (303) 602-5178
Facsimile: (303) 602-5184
Email: Emily.Caruso@dhha.org

Meggan Schmidt, BA

Telephone: (303) 602-5178
Facsimile: (303) 602-5177
Email: Meggan.Schmidt@dhha.org

Address for Correspondence:

Department of Emergency Medicine
Denver Health Medical Center
777 Bannock Street, Mail Code 0108
Denver, Colorado 80204 USA

PROTOCOL VERSION HISTORY AND AMENDMENTS

Date	Version Number	Description of Modifications
January 13, 2013	1.0	Included original quasi-experimental study design.
May 30, 2013	2.0	Changed design to randomized controlled trial.
July 30, 2013	2.1	Changed consent mechanism from opt-in to opt-out. Incorporate use of refined Denver HIV Risk Score (DHRS).
November 26, 2013	2.2	Amended exclusion criteria to include occupational HIV exposure.
March 20, 2014	2.3	Updated HIV testing methodologies and several methodological clarifications.
April 1, 2014	2.4	Amended exclusion criteria to remove prisoners.
January 29, 2015	2.5	Amended consent procedures to allow for waiver of consent for longitudinal follow-up.

ABBREVIATIONS AND DEFINITIONS

AIDS – Acquired Immunodeficiency Syndrome
BRST – Behavioral Risk Screening Tool
CDC – Centers for Disease Control and Prevention
CDPHE – Colorado Department of Public Health and Environment
CER – Cost-Effectiveness Ratio
CHIP – Children's Hospital Colorado Immunodeficiency Program
CI – Confidence Interval
COMIRB – Colorado Multiple Institutional Review Board
DHMC – Denver Health Medical Center
DHRS – Denver HIV Risk Score
ED – Emergency Department
EDWIN – Emergency Department Work Index
HAART – Highly Active Antiretroviral Therapy
HIV – Human Immunodeficiency Virus
ICER – Incremental Cost-Effectiveness Ratio
ILHE – Individual Level Health Education
IQR – Interquartile Range
IRB – Institutional Review Board
MSM – Men Who Have Sex with Men
PCM – Prevention Case Management
RR – Risk Ratio
SD – Standard Deviation
SFTP – Secure File Transfer Protocol
UCH – University of Colorado Hospital
USPSTF – United States Preventive Services Task Force
WHO – World Health Organization

CONTENTS

1. Specific Aims and Hypotheses 5

2. Background, Significance, and Rationale 5

3. Preliminary Studies 7

4. Methods 8

- **A. Study Design 8**
- **B. Settings 9**
- **C. Population 9**
- **D. Interventions 9**
- **E. Data Collection & Research Procedures 10**
- **F. Outcome Measures 12**
- **G. Data Management 13**
- **H. Analytic Plan 14**
- **I. Sample Size Estimation 15**

5. Human Subjects Protection 16

- **A. Description, Risks, and Justification of Procedures 16**
- **B. Estimated Duration of the Study 21**
- **C. Number and Distribution of Subjects 21**
- **D. Examinations, Laboratory Tests, Procedures and Follow-Up Visits 21**
- **E. Protected Health Information 22**
- **F. Risks 22**
- **G. Benefits 23**
- **H. Limitations 23**
- **I. Data Monitoring Plan 24**
- **J. Summarize Knowledge to be Gained 24**

6. References 25

APPENDIX

1. SPECIFIC AIMS AND HYPOTHESES

Early identification of undiagnosed HIV infection remains a critical public health priority. The research proposed in this project will evaluate the effectiveness of 3 rapid HIV screening methods, including a novel targeted strategy, in urban emergency department settings in the United States. The results of this study will help inform public health practices of how best to identify patients with whom to test for HIV infection in this important clinical setting, in an effort to improve the timeliness of diagnosis and linkage-to-care.

Specific Aim 1: To evaluate and compare the effectiveness of targeted and nontargeted rapid HIV screening strategies when fully-integrated into ED settings

Hypothesis 1a: Enhanced targeted rapid HIV screening (using the refined DHRS as a novel tool to identify high-risk patients) is significantly associated with new HIV diagnoses when compared to nontargeted rapid HIV screening.

Hypothesis 1b: Traditional targeted rapid HIV screening (using previously-recommended risk characteristics to identify high-risk patients) is significantly associated with new HIV diagnoses when compared to nontargeted rapid HIV screening.

Specific Aim 2: To measure and compare programmatic costs associated with implementation of targeted and nontargeted rapid HIV screening strategies when fully-integrated into ED settings

Hypothesis 2: Enhanced and traditional targeted rapid HIV screening is more cost effective per newly-identified HIV-infected patient than nontargeted rapid HIV screening from the institutional perspective.

Specific Aim 3: To measure and compare ED operational processes and crowding when targeted and nontargeted rapid HIV screening strategies are fully-integrated into ED care

Hypothesis 3: Enhanced targeted rapid HIV screening is not associated with: **(a)** longer patient waiting times; **(b)** longer patient length of stay times; **(c)** longer patient ED boarding times; **(d)** larger proportion of patients who leave prior to completing medical evaluation; or **(e)** greater overall crowding when compared to traditional targeted rapid HIV screening or nontargeted rapid HIV screening.

2. BACKGROUND, SIGNIFICANCE, AND RATIONALE

In the United States, over 1.1 million individuals are infected with HIV, approximately 250,000 remain undiagnosed, and 50,000 new infections occur annually.[1,2] These estimates have not changed dramatically over the last 15 years and new diagnoses appear to be on the rise in certain populations. Although the largest group of persons newly-infected remains men who have sex with men (MSM), new cases continue to be found disproportionately in racial/ethnic minorities, adolescents, and young adults.[1,2] Undiagnosed HIV infection continues to be disproportionately represented in the ongoing epidemic and its early identification remains a critical public health priority.

Testing for HIV infection is the first in a series of important interventions aimed at impacting the epidemic. Identifying individuals with HIV infection provides a critical opportunity to link them into care where treatment with antiretroviral medications reduces viral loads, thus slowing disease progression and reducing infectivity.[3,4] Also, knowing one's serostatus attenuates individual behaviors that contribute to transmission of the virus.[5] Between 2006 and 2011, the Centers for Disease Control and Prevention (CDC) and the White House released numerous recommendations and strategies in an effort to increase testing rates and, therefore, identification of undiagnosed HIV infection. The CDC strongly recommends

nontargeted opt-out HIV screening in all healthcare settings where the undiagnosed prevalence is≥0.1%, and the National HIV/AIDS Strategy recommends prevention methods that combine "… scientifically proven, cost-effective, and scalable interventions targeted to the right populations in the right geographic areas…".[6-10] High impact prevention prioritizes effectiveness and costs, feasibility of implementation, and coverage of target populations. In this context, HIV testing remains the principal means of secondary prevention of HIV infection.

Emergency departments (EDs) are a major focus of HIV testing efforts in the United States,[11] prompted by the fact that over 120 million ED visits occur annually,[12] they serve substantial numbers of underserved patients,[13] and are the most common site of missed opportunities for diagnosing HIV infection.[14] Given these features, EDs are an ideal setting to identify patients with undiagnosed HIV infection. Recognizing its potential value, in 2001 the CDC endorsed ED-based HIV screening as part of the national strategy to contain HIV infection;[15] unfortunately, several challenges remain. From 1993 through 2005, only approximately 0.4% of all ED patients had HIV testing performed.[16] With more aggressive initiatives, including the 2006 CDC recommendations, and several feasibility studies,[17-27] a larger number of EDs in the United States have incorporated HIV testing into routine practice.[28] However, most EDs still rely primarily on diagnostic testing (74% in academic EDs and 62% in non-academic EDs) with relatively sparse uptake of nontargeted screening (16% in academic EDs and 6% in non-academic EDs).[29]

In 2007, Rothman *et al.* published a conceptual framework of ED-based HIV testing strategies.[11] Within this framework, the following 3 HIV testing approaches were described: (1) diagnostic testing (defined as testing performed by a clinician based on clinical signs or symptoms); (2) targeted screening (defined as testing high-risk subpopulations based on identification of patient characteristics associated with HIV infection); and most recently; (3) nontargeted screening (defined as testing all individuals regardless of risk).[30]

Diagnostic testing is an important method of identifying patients with HIV infection in the ED.[23] This approach, introduced by the CDC in 1986,[31] is inherently understood by clinicians because of its potential to directly impact patient care. However, even when such testing is available, many clinicians do not maintain a sufficiently high suspicion for HIV infection among their patients, thus dramatically limiting the effectiveness of this approach.[23] Targeted screening allows for risk-based testing of patients, a prevention approach most would consider intuitive when attempting to impact an epidemic. This approach, however, relies on the assumptions that characteristics of the epidemic are fully appreciated in the targeted population, identifying patients who are at risk is possible, and sufficient resources are available to perform broader testing.[32] Nontargeted screening, on the other hand, was recommended in an effort to provide all patients, regardless of risk, the opportunity to be tested for HIV infection. Implementation of such large preventive interventions in busy EDs has proven difficult, with success limited to relatively few interested institutions with dedicated resources.[33] Since 2006, 11 studies have evaluated nontargeted HIV screening in an ED setting. Although all have demonstrated the ability to identify patients with HIV infection, the effectiveness of such screening can best be judged as modest.[34]

In 2007 the World Health Organization (WHO) released its guidance on provider-initiated HIV testing in healthcare facilities, and in contrast to the 2006 CDC recommendations, recommended more selective HIV testing (i.e., diagnostic testing or targeted screening) in settings where the HIV epidemic was concentrated.[35] According to WHO definitions, even the highest prevalence areas in the United States are considered concentrated. Similarly, in 2007, the United States Preventive Services Task Force (USPSTF) recommended targeted HIV screening as the principal approach to HIV testing, based in part, on the assessment that little empiric evidence existed to support a broader HIV screening initiative.[36] More recently, the USPSTF revised their recommendations to state, "clinicians [should] screen for HIV infection in adolescents and adults aged 15 to 65;" however, in their document, the Task Force does not explicitly

define how screening should be performed.[37] Although there is now apparent synergy between the CDC, USPSTF, and the National HIV/AIDS Strategy, relatively little has been done to understand which screening approach in EDs is most effective and efficient, especially when resources are constrained. Critical barriers to progress in this field remain and result from the paucity of empiric evidence to drive practice change. While research has demonstrated ED capacity to identify unrecognized HIV infection, limited research has compared clinical outcomes, programmatic methods, or cost effectiveness of different HIV screening approaches and their impact on ED services. Public health recommendations, including those on HIV screening, must be rooted in the results of rigorous, large-scale, comparative effectiveness research. Recognizing the practical limitations of "routine screening" in clinical settings is important,[38] while striving to balance efficient resource utilization with effective patient selection and testing methods.[39] Expanded testing programs is important, although further research is necessary to prioritize effectiveness and costs, feasibility of implementation, and coverage of target populations.[9] What remains unknown is whether nontargeted HIV screening is the optimal method of identifying patients with HIV infection or whether other approaches (e.g., targeted strategies) may be more effective.[40] This project will address these knowledge gaps with high-quality effectiveness research, and will provide the basis for how HIV screening should be performed in ED settings.

3. PRELIMINARY STUDIES

Our research team has pioneered investigations in ED-based HIV testing since 2004, and recent work has impacted HIV testing practices in the United States.[41-43] The overarching goal of our work is to evaluate HIV testing models in the ED and to combine scientific rigor with program evaluation to best inform processes and policies on a national level.[44] Through a series of projects we have determined that:

1) Although nontargeted opt-out rapid HIV screening is associated with newly-diagnosed HIV infection in the ED, the absolute number of patients identified is modest and most patients are identified late in their disease course. A large number of HIV tests have to be performed to identify a relatively small number of new HIV diagnoses, suggesting that many patients are missed with this approach, findings which have recently been confirmed by others.[34] We also conclude that nontargeted screening does not adversely affect ED processes of care, and is more costly per new diagnosis than diagnostic testing.[45,46]

2) When integrating nontargeted rapid HIV screening into a computerized kiosk system during triage, a large number of patients agree to rapid HIV testing when using an opt-out consent method in an ED setting; however, a larger proportion of those who accepted testing actually completed testing when an opt-in approach was used. Furthermore, and perhaps most importantly, patient understanding of HIV screening using opt-out consent is poor when compared to opt-in consent, and that the use of opt-in consent should be considered the approach for obtaining consent because it results in very little misunderstanding.[47] Despite this, in order to be consistent with CDC recommendations, we will use an opt-out consent approach in this study with added consent confirmation to mitigate patient misunderstanding.

3) The use of an empirically-derived, externally-validated risk-based instrument, the Denver HIV Risk Score (DHRS), accurately categorizes patients into groups with increasing probability of HIV infection,[48] and when implemented in an urban ED setting is associated with new HIV diagnoses when compared to nontargeted screening.[49] In a single-center pilot comparison of the DHRS to nontargeted screening, we found that while both HIV screening methods identified the same absolute number of newly-diagnosed patients, significantly fewer tests were required when using the DHRS to target patients for screening. [49] The DHRS was refined further and externally validated using a national HIV testing cohort. In this study two items related to specific sexual behavior (receptive anal intercourse and

vaginal intercourse) were removed in order to create a more succinct and easier to implement risk assessment tool. This refined DHRS performed similarly to the original DHRS.[50]

4. METHODS

A. Study Design

Specific Aim 1

For this aim, we will perform a multi-center, prospective, randomized, controlled clinical trial to evaluate the effectiveness of three distinct HIV screening approaches. Implementation of a randomized controlled trial will incorporate use of "pragmatic" or effectiveness methodologies,[51-53] and will allow us to compare the effectiveness of the three screening approaches while minimizing threats to internal validity. Patients will be screened using one of three interventions in a 1:1:1 random sequence; patients will therefore be offered HIV testing based on the results of which screening arm they are assigned, and in the case of the two screening tools, the results of risk assessment. All screening methodologies will be integrated into electronic patient triage systems at each site; as such, the actual randomization process will also be integrated into the electronic system, allowing for real-time concealed random allocation (**Figure 1**). Nurses who perform screening and all ED staff (e.g., physicians, nurses, and technicians) will understand the conceptual goals of the project but will be blinded to the specific hypotheses, and patients will be completely blinded to the purpose of the study.

Figure 1. Study schema. (*Inclusion: ≥16 years of age; clinically stable; capable of providing consent for general medical care; Exclusion: self-identified as infected with HIV; victim of a sexual assault; occupational HIV exposure; Fast Track (time in ED is expected to be <30 minutes). Abbreviations: BRST=Behavioral Risk Screening Tool; DHRS=Denver HIV Risk Score.)

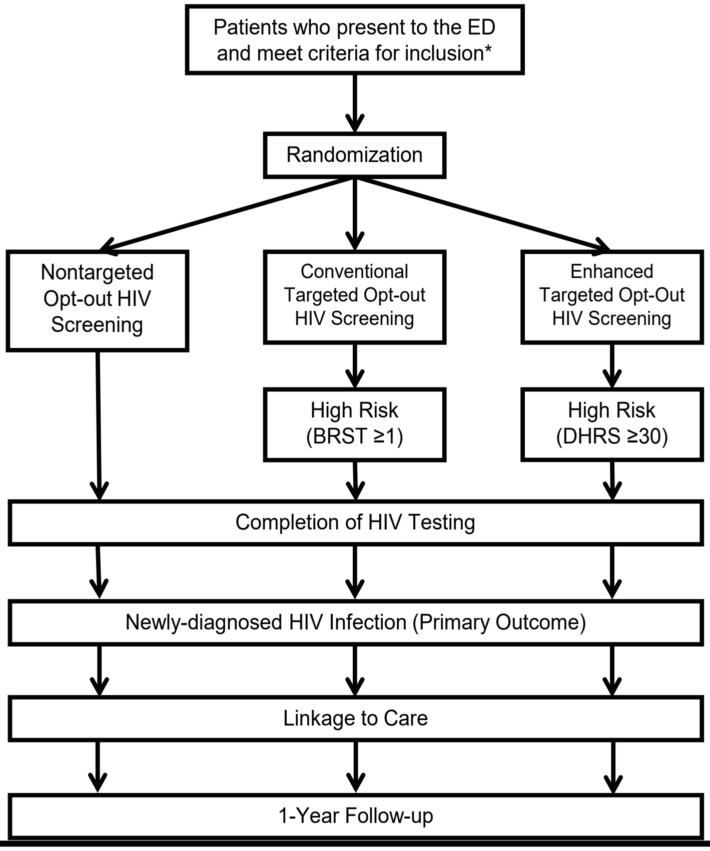

All of the participating sites have established ED-based HIV testing programs, and all of the testing procedures that occur after patients are identified for HIV testing (i.e., consent for the test, performance of the test, results disclosure, linkage to care, etc.) will have been integrated into clinical processes within each ED as standards of care. The specific interventions being evaluated in this study relate only to how patients are identified for and offered HIV testing. The standard process used by the DHMC ED and the integration of the three study interventions is described by the "ED Rapid HIV Screening Workflow" figure (**Appendix**).

Specific Aims 2 and 3

For these aims, we will perform prospective cohort studies nested in the clinical trial described above.

B. Settings

This study will be performed in the EDs at: (1) Denver Health Medical Center (DHMC) as part of Denver Health and Hospital Authority in Denver, Colorado; (2) Highland Hospital as part of Alameda Health System in Oakland, California; (3) Johns Hopkins Hospital as part of Johns Hopkins University in Baltimore, Maryland; and (4) University of Cincinnati Medical Center in Cincinnati, Ohio. These sites were selected because of their heterogeneity of populations served, including a large proportion of underserved, racial and ethnic minorities, and adolescents; local HIV epidemics; geographic distributions in the United States; and expertise of investigators. DHMC will serve as the coordinating site for this project. However, each individual site will obtain approval from their respective Institutional Review Boards (IRBs). These approvals will be forwarded to the Colorado Multiple Institutional Review Board (COMIRB) when they are obtained.

C. Population

Criteria for Inclusion

Patients ≥16 years of age who present to the EDs during the study enrollment period will be eligible for inclusion if they are considered clinically stable by screening nurses or physicians and capable of providing consent for general medical care. Patients will be excluded from screening if they are: (1) <16 years of age; (2) unable to consent for care or HIV testing (e.g., altered mentation, critical illness or injury, etc.); (3) self-identified as infected with HIV; (5) victims of sexual assault; (6) presenting to the ED with occupational HIV exposure; or (7) eligible for the Fast-Track section of the ED where anticipated time in the ED is expected to be <30 minutes.

D. Interventions

We will perform a prospective clinical trial at four institutions across the United States.[51,52] Each study site will enroll consecutive eligible patients and randomize them to one of three rapid HIV screening interventions (described below), all of which are utilized as either local or national standards of care. The three screening interventions will be fully integrated into electronic patient screening and tracking systems in each ED and integrated into triage processes. Eligible patients will be screened by triage nurses in accordance with institutional standards of care; as part of this process, patients will be screened for HIV infection using one of the three screening tools in a 1:1:1 random sequence as determined by a random sequence generator built into the electronic patient screening systems. Patients will then be offered voluntary rapid HIV testing based on the screening arm they are assigned (i.e., patients randomized to the nontargeted HIV screening arm will be offered HIV testing without assessment of risk, whereas those randomized to the two targeted screening arms will be offered HIV testing only if they are identified as being at increased risk for HIV infection). To maximize generalizability to ED settings, screening will be performed 24 hours per day for the entire study enrollment period and all screening and testing methods will be fully integrated into ED operations.

Specific Aim 1

Intervention A: Nontargeted Rapid HIV Screening

This screening arm will consist of nontargeted rapid opt-out HIV screening. Nontargeted HIV screening is currently the recommended approach by the CDC.[6] Consecutive patients who present to the ED for evaluation, who meet criteria for inclusion, and are randomized to this arm will be offered voluntary, free,

and confidential rapid HIV testing by nurses using opt-out consent during medical screening. Patients who decline HIV testing during screening will be eligible for diagnostic HIV testing by their physicians.

Intervention B: Enhanced Targeted Rapid HIV Screening

This screening arm will consist of implementing targeted rapid opt-out HIV screening using the refined DHRS to identify patients at increased risk for HIV infection (**Table 1**). Prior to initiation of this study, this approach was the standard of care in the ED at DHMC. The refined DHRS will be incorporated into electronic medical screening and patient tracking systems of each ED. As such, nurses will be able to electronically enter responses to each of the risk score questions during screening. In addition, using methodology already developed in Denver,[49] the questions will be ordered to present potentially less sensitive questions before more sensitive questions, and each system will be developed to calculate a risk score real-time. A binary cut point was selected to define patients at increased risk (score ≥30) or not (score <30) based on results from our prior work and to simplify stratification when used by clinicians.[49] More recent national external validation work supports this as the appropriate threshold.[50] Patients identified as being at increased risk will be offered rapid HIV testing by nurses using opt-out consent. Patients identified as low risk will not be offered rapid HIV testing but will be eligible for diagnostic HIV testing by their physicians.

Intervention C: Traditional Targeted Rapid HIV Screening

This screening arm will consist of implementing targeted rapid opt-out HIV screening using conventional risk behaviors to identify patients at increased risk for HIV infection. A Behavioral Risk Screening Tool (BRST), adapted from HIV testing recommendations from the CDC and World Health Organization dating back to 1987,[15,35,56,57] will serve as a comprehensive list of behavioral risk associated with HIV infection (**Table 2**). This form of targeted HIV screening was previously recommended by the CDC prior to 2006 and the USPSTF, but has never been systematically evaluated in an ED environment or compared to other, more contemporaneous, HIV screening methods.[36] The BRST will be incorporated into the electronic medical screening and patient tracking systems in each ED using identical methodologies described for the refined DHRS (see Intervention B). Each of the 5 risk categories will be weighted equally so that the BRST score will range from 0 to 5. Nurses will apply the BRST to all patients who meet criteria for inclusion, and those who score ≥1 will be considered at increased risk for HIV infection and will be offered rapid HIV testing using opt-out consent. Patients who score 0 will be considered low risk for HIV infection and not offered rapid HIV testing; these patients will, however, be eligible for diagnostic HIV testing by their physicians.

Table 2. Behavioral Risk Screening Tool (BRST) for identification of HIV infection.

Please answer the following questions, "Have you ever…" OR if previously tested for HIV infection, "Since your last HIV test have you…"(Yes/No):

1. Injected drugs and shared equipment with others (e.g., needles, syringes, cotton, water)?
2. Had unprotected intercourse with someone that you think might be infected with HIV (e.g., a partner who injected drugs, been diagnosed with an STD or hepatitis, had multiple or anonymous sex partners, or exchanged sec for drugs or money)?
3. Had unprotected vaginal or anal intercourse with more than one sex partner?
4. Been diagnosed or treated for an STD, hepatitis, or tuberculosis?
5. Have you ever been told you have an infection related to a 'weak immune system'?

Any affirmative response of these questions is considered at increased risk for HIV.

Diagnostic Rapid HIV Testing and Other HIV Testing Components

Strengths of this study will be the inclusion of sites that use different implementation methods of HIV testing and linkage-to-care.[20,23,45,47,49,58,59] Several stipulations, however, will be required of each site, including: (1) implementation and full integration of the interventions and HIV testing processes into the EDs; (2) use of streamlined opt-out consent (but in accordance with state statutes); (3) use of Food and Drug Administration (FDA)-approved rapid HIV testing to ensure timely and valid result reporting; (4) additional performance of clinician-directed diagnostic HIV testing for those patients not screened or who were identified as low risk during either of the 2 targeted interventions, or who declined testing when offered during any of the screening intervention;[23] and (5) performance of confirmatory testing (using either Western blot or viral load assays) and appropriate linkage-to-care of those who test positive for HIV infection in the ED. Other operational features (e.g., which rapid tests are used, who performs prevention counseling, etc.) will not be explicitly stipulated during this study, although use of contemporaneous 4th generation HIV assays will be encouraged. Each institution has extensive experience performing rapid HIV testing in the ED and the methods that work at each site have been evaluated to ensure optimal patient care.

E. Data Collection & Research Procedures

<u>Specific Aim 1</u>

During this aim, we will collect the following data elements for all eligible patients: (1) patient visit information (unique patient identifier, acuity level, mode of arrival, and date/time of visit); (2) demographics (age, sex, race/ethnicity, primary language); (3) payer information (commercial, Medicare, Medicaid, self, or state-sponsored); (4) details of randomization, including the intervention assigned and results of risk screening, if applicable; (5) whether a patient was offered, accepted, and completed rapid HIV testing; (6) results from all rapid HIV tests; and for all patients with at least one reactive HIV test; (7) results of confirmatory test results, additional behavioral risk information as collected during post-test counseling, initial CD4 counts and viral loads, whether they were successfully linked into care, and details of follow-up care and disease progression. Data from (1) through (5) will be collected prospectively using methods developed and validated in Denver from each institution's electronic screening, patient tracking, and laboratory reporting systems.[61] Our study team has extensive experience interfacing with such systems and obtaining large amounts of valid patient-level data. Data from (6) and (7) will be retrospectively obtained using trained personnel and structured procedures. See **Data Dictionary**, **"Positive Patient Risk Information Data Collection Instrument"** and **"Longitudinal Data Collection Instrument" / "Limited Longitudinal Data Collection Instrument** in the **Appendix**.

<u>Specific Aim 2</u>

During this aim, we will include randomly-selected time periods for which patients are included in this aim in an effort to ensure unbiased sampling. Direct program costs related to training, education, and implementation will be collected for each intervention at each study site. Program costs will include: (1) startup (computers, software, programming, and training); (2) personnel (administrative, ED, laboratory staff, and prevention counselors); and (3) supplies and equipment (informational sheets, HIV test kits, and blood draw supplies). Non-personnel costs will be obtained through expense reports. Personnel costs will be determined by trained research assistants by identifying position titles of those involved in each part of the interventions and collecting time-motion data using a structured data collection instrument. Time-motion data will be collected during randomly-selected time periods during patient enrollment in an effort to ensure unbiased sampling. Trained research assistants will observe all processes of HIV screening and record time (in seconds) to complete each component. To estimate costs associated with performing

these interventions in an ED environment, we will also specifically include nights and weekends, and will specifically collect patient and staff data, stratified by study arm in order to ensure appropriate time measurements by intervention. Time per patient will be translated into time per month and per year, by position title, and position titles will be linked to median salary data from the Bureau of Labor Statistics for the respective metropolitan statistical area.[54] See **Data Dictionary** and **"Time Motion Study Data Collection Instrument"** in the **Appendix**.

Specific Aim 3

In this aim, we will collect additional ED process data in conjunction with patient enrollment as part of the clinical trial described in Specific Aim 1. We will include data from all patients enrolled in Specific Aim 1. Values for each of the four basic time metrics (patient waiting time, patient length of stay, patient boarding time, and proportion of patients who leave before completion of evaluation) will be collected real-time for all enrolled patients evaluated in each ED using electronic patient tracking systems. Patients who register for evaluation in each ED on a daily basis include time stamps related to initiation of medical screening, ED bed assignment, patient discharge or admission to the hospital and, if admitted to the hospital, inpatient bed assignment. The fifth metric, ED crowding, will be calculated using one of two validated ED crowding metrics (described in detail below). See **Data Dictionary** in the **Appendix**.

F. Outcome Measures

Specific Aim 1

Confirmed newly-diagnosed HIV infection will serve as our primary outcome. In anticipation of testing patients with previously diagnosed HIV infection (i.e., repeat diagnoses),[45] our secondary outcome will include a composite of new diagnoses and repeat diagnoses.

Although confirmed newly-diagnosed HIV infection will serve as the primary outcome for this study, we believe important secondary effectiveness evaluations include disease state at the time of diagnosis as well as a longer-term evaluation of access to care, disease progression, and treatment. Therefore, additional outcomes will include behavioral risk information, CD4 count (cells/μL) and viral load (copies/mL) at the time of diagnosis, and successful linkage into care (defined as completion of an initial HIV clinic visit). Details of follow-up care and disease progression for a period of one year following diagnosis will also be collected, including the number of scheduled and unscheduled medical care visits, whether antiretroviral therapy and treatment for opportunistic infections were initiated, the number of hospitalizations, and mortality.

Specific Aim 2

Programmatic costs associated with implementation of targeted and nontargeted rapid HIV screening strategies when fully-integrated into ED settings will be measured. We will estimate program costs using a simple cost allocation method that sums fixed and variable costs for program implementation. Components of costs by intervention arm will include: start-up, personnel, tests, supplies, and equipment. In order to estimate personnel-related costs by arm, time-motion data will be collected during randomly-selected time periods of the trial at each site (including different times of day and weekends). Personnel time spent per patient will be monetized by position title-specific median salary data from the Bureau of Labor Statistics for the respective metropolitan statistical area.[54] Total programmatic costs, costs per person tested, and cost per HIV case detected will be compared by intervention arm. Differences across sites will be explored in sensitivity analyses. Uncertainty in cost estimates will be estimated using bootstrapping resampling methods.[54]

Specific Aim 3

Five specific metrics of ED patient throughput will be measured in this aim. All five measures are commonly thought to represent ED processes of care and crowding. The first metric, patient waiting time, will be defined as the time from initiation of medical screening by the screening nurse to the time the patient is assigned a bed in the ED. The second metric, patient length of stay, will be defined as the time from when the patient is assigned a bed in the ED to the time of discharge or, for those admitted to the hospital, the time an inpatient bed is requested by a physician. The third metric, patient boarding time, will be defined as the time from when an inpatient bed is requested to the time the patient leaves the ED for that bed. The fourth metric, proportion of patients who leave before completion of evaluation, will be defined as the proportion of all patients who either leave before being placed in a treatment room or leave before completion off their evaluation. The fifth metric, Emergency Department Work Index (EDWIN)[55] or the National ED Overcrowding Score (NEDOCS),[62] are global or composite crowding metrics and are defined as:

$$EDWIN = \sum n_i t_i / N_a(B_T - B_A),$$

where n_i = the number of patients present in the ED by triage category (i); t_i = the acuity category (defined using an ordinal scale from 1 to 5, 1 being the most acute and 5 being the least acute); N_a = the number of physicians on duty at a given time; B_T = the total number of beds available in the ED; and B_A = the number of admitted patients waiting for inpatient bed assignment in the ED. The EDWIN metric can range from 0 to infinity, and a previous validation study demonstrated median EDWIN scores of 1.1 (IQR: 0.8 – 1.6), 1.6 (IQR: 1.2 – 1.9), and 1.8 (IQR: 1.4 – 2.5) during non-busy, average, and busy ED time periods;[55] or

$$NEDOCS = -20 + 85.8(\text{total ED patients} \div \text{total ED beds}) + 600(\text{boarded patients} \div \text{total hospital beds}) + 13.4(\text{number of ventilated patients in the ED}) + 0.93(\text{longest boarder time in hours}) + 5.64(\text{longest time for patient waiting in hours})$$

where 0 – 20 is considered “not busy”, 21 – 60 considered “busy”, 61 – 100 considered “very busy”, 101 – 140 considered “crowded”, 141 – 180 considered “dangerous”, and 181 or greater considered “disaster”. EDWIN and NEDOCS have been compared head-to-head and have both been shown to be valid and comparable metrics for quantifying ED crowding.[63]

Values for each of the four basic time metrics will be collected real-time for all patients enrolled into the study using electronic patient tracking systems. Patients who register for evaluation in each ED on a daily basis include time stamps related to initiation of medical screening, ED bed assignment, patient discharge or admission to the hospital and, if admitted to the hospital, inpatient bed assignment. Because EDWIN and NEDOCS rely on point-in-time sampling and at the level of the ED, it is not feasible to measure their variables on a continuous, real-time basis. However, we will calculate the fifth ED metric, either EDWIN or NEDOCS, at the time each patient begins medical screening and when he or she is discharged. The difference between these data will be used as a covariate in the analyses for this aim as described below.

G. Data Management

For all aims, data will be electronically transferred or manually entered into a secured electronic database (Microsoft SQL, Microsoft Corporation, Redmond, WA) or spreadsheet (Microsoft Excel, Microsoft Corporation, Redmond, WA). The Denver site will serve as the Data Coordinating Center (DCC) and data from the three non-Denver institutions will be transferred to the DCC using a secure file transfer protocol (SFTP). Data will be transferred, concatenated, and cleaned using SAS (SAS Institute, Inc., Cary, NC), and statistical analyses will be performed using SAS Version 9.3, Stata Version 12 (Stata Corporation,

College Station, TX), or their most current versions (for all Specific Aims), and Microsoft Excel (for Specific Aim 2). See Data Protection section below for additional details related to data acquisition, cleaning, and transfer to the DCC.

H. Analytic Plan

All statistical analyses will be conducted by the Principal Investigator and independently confirmed by the study's biostatistician blinded to study allocation. Final analyses will be performed after cleaning and locking of the dataset. Although no formal interim analyses are planned, the study team may perform preliminary analyses for purposes of presentation at scientific meetings; these instances, if they occur, will be explicitly described as preliminary and qualified as such.

<u>Specific Aim 1</u>

Descriptive analyses will be performed for all variables. Continuous data will be reported as means with standard deviations (SDs) or medians with interquartile ranges (IQRs), depending on whether they are normally distributed or non-normally distributed, respectively; additionally, categorical data will be reported as proportions or percentages with 95% confidence intervals (CIs). Bivariate statistical tests (e.g., Student's t-test or Wilcoxon rank sum test for two-group parametric or non-parametric comparisons, ANOVA or Kruskal-Wallis test for three-group parametric or non-parametric comparisons, and chi-square test or Fisher's exact test) will be used to compare variables between study groups. Patient-level data will be reported for demographics. Otherwise, patient-visit data will be reported given the relatively large degree of recidivism to EDs; as such, the primary unit of analysis will be patient visits.

All analyses will be performed using the intention-to-treat principle and no interim analyses are planned given the "pragmatic" trial approach and minimal risk to subjects. Given the randomized controlled trial design, the <u>primary comparison</u> will include unadjusted risk ratios (RRs) for newly-identified HIV infection (primary outcome) with 95% CIs, specifically comparing enhanced targeted HIV screening to nontargeted HIV screening (the primary hypothesis of this study). Statistical significance for the primary analysis will be defined as <0.05 based on two-tailed statistical testing, which includes a lower 95% confidence limit of >1.0.

<u>Secondary comparisons</u> will include unadjusted RRs for: (1) traditional targeted HIV screening to nontargeted HIV screening (the secondary hypothesis of this aim); and (2) the composite secondary outcome, newly-diagnosed and repeat-diagnosed HIV infection for both hypotheses. Additionally, HIV prevalence, including overall prevalence and test prevalence, and 95% CIs will be reported for each study group, and institution-specific subgroup analyses will be performed as well as hierarchical multivariable binary Poisson regression to account for clustering at the level of the institution and repeat visits at the level of the patient, while adjusting for age, sex, race/ethnicity, primary language, pay status, and acuity level. Results of these latter analyses will be reported as adjusted RRs with 95% CIs. Finally, additional comparative analyses will be performed, including: (1) absolute numbers of new HIV diagnoses; (2) proportions identified early in the disease course (defined by an initial CD4 >350 cells/µL) and late in the disease course (defined by an initial CD4 <200 cells/ µL); (3) proportions successfully linked into care; (4) patient characteristics stratified by those who do and do not agree to HIV testing after screening; and (5) frequency of unscheduled medical visits, use of antiretroviral therapy, treatment for opportunistic infections, hospitalizations, and mortality over the 12 months following initial diagnosis. Any other analyses will be considered exploratory and described as post-hoc.

Specific Aim 2

All cost and time-motion data will be manually entered and analyses will be performed using Microsoft Excel (Microsoft Corporation, Redmond, WA). Double data entry will be performed to minimize data entry error. Total direct costs will be calculated and compared between interventions. As each intervention will not extend beyond 12-months, it will be unnecessary to discount costs, although all costs will be annualized. We will use the number of newly-diagnosed HIV-infected patients, the primary outcome from Specific Aim 1, as an intermediate outcome for this aim.[64] The scope of this aim is not to perform decision analyses or disease transition-state modeling,[65] but instead to systematically collect valid cost data and to report cost effectiveness of the 3 screening approaches using an intermediate outcome. Results of this aim will inform future modeling, including from a society perspective, by our team. Total direct costs per patient identified with HIV infection at the institutional level will be reported as CERs and incremental CERs, or the additional costs per patient identified with HIV infection beyond those incurred by nontargeted screening. Because underlying distributions of cost data are often skewed, we will report bootstrap 95% CIs for each CER and ICER.[66] Finally, sensitivity analyses will be performed to determine the effects of individual parameter ranges on the results.[67]

Specific Aim 3

Process metric variables for all enrolled patients will be compared between study arms. Because the time and EDWIN or NEDOCS metrics are continuous, they will be reported as means with SDs or medians with IQRs. Bivariate analyses will be used to estimate statistical differences between each process metric and the three HIV screening interventions. Because we propose measuring five ED process metrics, we have defined statistical significance for this part of the analyses to be a Bonferroni-adjusted p-value <0.01. Also, hierarchical multivariable linear regression analyses will also be used to estimate associations between each of the interventions and continuous process metrics, while accounting for clustering at the institution level and adjusting for overall ED crowding as measured by the difference in either EDWIN or NEDOCS for the duration of the patient's visit. If necessary, continuous data will be transformed and multiple imputation will be used in instances where data are missing.[68-70] The primary unit of analysis will be patient visits.

I. Sample Size Estimation

The primary hypothesis of this study is that enhanced targeted HIV screening will be significantly associated with new HIV diagnoses when compared to nontargeted HIV screening. As described above, the primary statistical measure will include an unadjusted RR. Based on our *a priori* assumptions related to the performance of each screening method weighted across the four study sites (i.e., nontargeted screening – 25% HIV test completion, 0.3% HIV test prevalence; enhanced targeted screening – 5% HIV test completion, 2% HIV test prevalence), we anticipate requiring a minimum of 12,600 HIV tests performed to achieve a statistically significant association using an alpha of 0.05 (i.e., estimated unadjusted RR = 1.33, 95% CI: 1.01-1.79). We also anticipate adjusting variance estimates to account for within-institution correlation; as such, we will plan to complete at additional 10%, resulting in a total of 14,000 HIV tests performed.

5. HUMAN SUBJECTS PROTECTION

A. Description, Risks, and Justification of Procedures

Recruitment Methods

All patients who present to the ED during the study periods, and who meet eligibility criteria, will be included. Patients who meet eligibility criteria will be randomized to one of three screening interventions that will be integrated into the electronic patient tracking systems. Randomization will occur automatically during implementation of standard-of-care triage via programming integrated into the electronic patient tracking and triage systems. Eligible patients assigned to the nontargeted HIV screening arm will be offered on-site, voluntary, and confidential rapid HIV testing by nurses using opt-out consent during medical screening (triage) examination. Patients assigned to the enhanced targeted HIV screening arm will be asked the refined DHRS questions during the medical screening (triage) examination. Patients in this group who are categorized as “high risk” (defined as a refined DHRS score ≥30) will be offered on-site, voluntary, and confidential rapid HIV screening by nurses using opt-out consent. Patients who are assigned to the traditional targeted HIV screening arm will be asked the BRST questions during the medical screening (triage) examination. Patients in this group who are categorized as “high risk” (defined as a BRST score ≥1) will be offered on-site, voluntary, and confidential rapid HIV screening using opt-out consent. Patients assigned to the two targeted HIV screening arms and identified as “low risk” (defined either as a refined DHRS ≤30 or a BRST score = 0) will not be routinely offered rapid HIV testing; however, they will be eligible for diagnostic HIV testing by their treating physicians. Similarly, patients assigned to the nontargeted HIV screening arm but who decline HIV testing will also be eligible for diagnostic HIV testing by their treating physicians.

During study enrollment, an HIV informational form about HIV infection will be made available to patients (see **“Denver Health Information about HIV”** in the **Appendix**). The Denver Health informational sheet will be adapted for each study site. Also, during the entire study period, all patients who test positive for HIV infection will undergo structured posttest counseling by dedicated clinical social workers. These patients will be offered the opportunity to participate in the follow-up portion of this study (see below). All staff offering testing to patients will have a treatment relationship with the patients. Women and minorities will be included in this study without exception.

Consent Procedures

All patients who present to the ED at DHMC during this project will receive standard-of-care medical evaluation and treatment and may be asked questions about their risk for HIV infection, using either the refined DHRS or the BRST instruments, and may be offered, as voluntary and routine practice, rapid HIV testing using an opt-out mechanism. Consent for rapid HIV testing will be documented in the patient’s medical record, as it is currently done as part of standard of care. The medical care for those patients who do not receive rapid HIV testing will not vary from those who do, except that the physician will know each patient’s HIV test result and may alter the medical evaluation based on this additional information. Because rapid HIV testing is a standard-of-care in the ED at DHMC (and the other study sites) and because this project will be evaluating three processes for performing HIV screening in this clinical setting (i.e., nontargeted vs. two targeted screening methods), this potential change in care is consistent with current medical practice.

We are requesting a waiver of consent for everyone included as part of the evaluation of the three HIV screening strategies. This request for waiver is based on the following in accordance with 45_CFR_46.116(d)(1-4):

(1) This part of the study is minimal risk because the only risks include breach of confidentiality and possible risks of change from standard of care (although each screening method is consistent with recommended national or local standards of care). The most significant risk to patients will be loss of confidentiality, which is viewed as minimal. All of the methods utilized in this study are currently accepted, recommended and utilized methods for HIV screening, locally or nationally. Furthermore, there is minimal difference in risk between the 3 screening interventions. While there is a potential risk associated with missed opportunities for HIV diagnosis, we believe that this potential risk is also minimal. We also believe that clinical equipoise exists in that there is no empiric evidence that one HIV screening method is superior than another despite policy statements (e.g., CDC and USPSTF) that suggest otherwise; the effectiveness of HIV screening is even more uncertain in ED settings. All medical centers in Colorado, including Denver Health Medical Center, are required by state law to report all diagnoses of HIV infection to the state health department. Patients who access testing services in routine health care settings are exposed to this same risk. Therefore, the risks involved in this study are no more than what the patient would experience in standard care. The Principal Investigator will assume full responsibility for the protection of all study-related documents and datasets, including those that contain protected health information. The Project Coordinator and Principal Investigator will oversee all data collection. All electronic data will be held in an electronic database (Microsoft SQL, Microsoft Corporation, Redmond, WA) using the protected network at DHMC. The database will be password protected and stored in the Project Coordinator's network user a separate folder on the DHMC mainframe network. This network includes firewall protection and the folder can only be accessed by members of the study team. All paper documents will be stored in a locked file cabinet in the Project Coordinator's office. These data will be manually entered into the main electronic database. During this project, all data will be kept in secure locations either in the ED, the office of the Principal Investigator, the office of the Project Coordinator, the office of the clinical social workers, or in the laboratory;

(2) It does not violate patients' rights (the research will offer HIV testing by asking standard-of-care questions but the patient will still be able to decide whether HIV testing occurs). The risks involved in this study are no more than what the patient would experience in standard care and each patient will be able to decide whether HIV testing occurs**;**

(3) Separate written informed consent cannot practicably be carried out without a waiver because of the large number of consecutive patients included from a busy ED, and the potential for biased participation in the study. It is essential that we have 100% participation in this study in order to identify the rate of newly-diagnosed HIV infection, which is the primary outcome for this study. We believe this waiver of consent will not adversely affect the rights and welfare of the subjects involved in this project, as they will only be subjected to current standards of care and they will all have the right to refuse HIV testing ; and

(4) Each patient will have an experience that does not differ significantly from usual care.

We will obtain written informed consent from all patients who test preliminarily positive for HIV infection in the ED (this number will represent approximately 0.5% of all patients tested for HIV infection, and less than 0.1% of all ED patients overall) in order to obtain additional data including specific behavioral risks and data regarding follow-up with medical and preventive care referrals for up to 1 year following

diagnosis. During posttest counseling, the clinical social workers, all of whom will have completed IRB training, will obtain this consent. This process will take place in a quiet and unhurried setting in the patient's room. The patient will be given enough time to ask questions about the collection of follow-up data. The patient's comprehension will be assessed by asking the patient to describe the follow-up procedures in their own words and a copy of the executed consent form will be provided to the patient. If limited chart abstraction will be performed in lieu of a more detailed patient contact and chart review, a waiver of consent will be obtained for the longitudinal follow-up portion of the study and chart abstraction will be conducted retrospectively.

<u>Special Consent Issues</u>

As described in "Consent Procedures", we are requesting that separate research consent not be obtained for this study. Separate research consent will be obtained for the follow-up portion of the study if applicable. Pregnant women and prisoners will not be excluded from this study. Pregnant women and prisoners are routinely tested for HIV infection at all study sites.

HIV Testing Consent Process (Standard of Care)

Patients who are ≥16 years of age will also not be excluded; therefore children may be potentially enrolled in this study. Under current laws in Colorado, California, Maryland, and Ohio (see below), minors are allowed to consent for HIV testing and treatment and other sexually transmitted infections without parental consent. To be consistent with these laws, patients who are under the age of 18 years of age and agree to rapid HIV testing during this study will be able to do so without parental consent as standard of care.

<u>Colorado State Section Code §25-4-402 (4)</u>

Any physician, upon consultation by a minor as a patient and with the consent of such minor patient, may make a diagnostic examination for sexually transmitted infection and may prescribe for and treat such minor patient for sexually transmitted infection without the consent of or notification to the parent or guardian of such minor patient or to any other person having custody of or parental responsibilities with respect to such minor patient.

<u>Colorado Revised Statute §25-4-1405 (6)</u>

(6) Any local health department, state institution or facility, medical practitioner, or public or private hospital or clinic may examine and provide treatment for HIV infection for any minor if such physician or facility is qualified to provide such examination and treatment. The consent of the parent or guardian of such minor shall not be a prerequisite to such examination and treatment. The physician in charge or other appropriate authority of the facility or the licensed physician concerned shall prescribe an appropriate course of treatment for such minor. The fact of consultation, examination, and treatment of such a minor under the provisions of this section shall be absolutely confidential and shall not be divulged by the facility or physician to any person other than the minor except for purposes of a report required under sections 25-4-1402 and 25-4-1403 and subsection (8) of this section and a report containing the name and medical information of the minor made to the appropriate authorities if required by the "Child Protection Act of 1975", part 3 of article 3 of title 19, C.R.S. If the minor is less than sixteen years of age or not emancipated, the minor's parents or legal guardian may be informed by the facility or physician of the consultation, examination, and treatment. The physician or other

health care provider shall counsel the minor on the importance of bringing his parents or guardian into the minor's confidence about the consultation, examination, or treatment.

Maryland State section code §20-102 (c3)

A minor has the same capacity as an adult to consent to: (1) treatment for or advice about drug abuse; (2) treatment for or advice about alcoholism; (3) treatment for or advice about venereal disease.

California State section code §121020

(a) (1) When the subject of an HIV test is not competent to give consent for the test to be performed, written consent for the test may be obtained from the subject's parents, guardians, conservators, or other person lawfully authorized to make health care decisions for the subject. For purposes of this paragraph, a minor shall be deemed not competent to give consent if he or she is under 12 years of age.

California State section code §6926

(a) A minor who is 12 years of age or older and who may have come into contact with an infectious, contagious, or communicable disease may consent to medical care related to the diagnosis or treatment of the disease, if the disease or condition is one that is required by law or regulation adopted pursuant to law to be reported to the local health officer, or is a related sexually transmitted disease, as may be determined by the State Public Health Officer.

Ohio State section code §3701.242

(A) An HIV test may be performed by or on the order of a health care provider who, in the exercise of the provider's professional judgment, determines the test to be necessary for providing diagnosis and treatment to the individual to be tested, if the individual or the individual's parent or guardian has given consent to the provider for medical or other health care treatment. The health care provider shall inform the individual of the individual's right under division (D) of this section to an anonymous test.

(B) A minor may consent to be given an HIV test. The consent is not subject to disaffirmance because of minority. The parents or guardian of a minor giving consent under this division are not liable for payment and shall not be charged for an HIV test given to the minor without the consent of a parent or the guardian.

Follow-Up Research Consent Process

As described above, patients who are ≥16 years of age will not be excluded; therefore children may be enrolled in this study, agree to HIV testing, and be diagnosed with HIV infection. Under current laws in Colorado, California, Maryland, and Ohio, minors are allowed to consent for HIV testing and treatment without parental consent. Thus, we are requesting patient assent (without parental consent) for those under the age of 18 years who test positive for HIV infection in the ED for the collection of follow-up data relating to HIV treatment. This will be obtained using a written assent form. In cases where study sites choose to do limited chart abstraction in lieu of a more detailed chart abstraction, a waiver of consent will be obtained for the longitudinal portion of the study.

Authorization Procedure

We are requesting a waiver of authorization for all patients initially included in this study. Authorization for the collection and use of protected health information (PHI) will be obtained, however, by using the routine authorization forms utilized in the ED at DHMC as part of standard medical care. Data collected as part of this project, including all patients who seek care in the ED over the five-year study period, will be no more than a minimal risk to privacy, and HIPAA Authorization cannot be practicably carried out without a waiver due to the large number of patients included and because its requirement may bias participation. In addition, this research cannot be done without specific requested PHI.

However, we will obtain HIPAA Authorization as part of the follow-up portion of this study. This authorization will be obtained from all patients who test preliminarily positive for HIV infection during their ED visits. Authorization will be obtained during the written informed consent or assent process by clinical social workers or other study personnel using approved consent form templates that include HIPAA Authorization. In cases where study sites choose to do limited chart abstraction in lieu of a more detailed chart abstraction and possible direct contact with the patient or his/her clinician, a waiver of HIPAA authorization will be requested. This project meets the requirements for a waiver of HIPAA authorization for the same reasons that requirements for a waiver of consent are met.

Data Protection

Each study site will follow the same data collection and transfer procedures to ensure maximal confidentiality of all patients. At each site, investigators will compile the following data, either directly from the ED information systems or from individual data collection instruments designed specifically for this study: (1) patient visit information (name, medical record number, acuity level, mode of arrival, and date/time of visit); (2) demographics (age, sex, race/ethnicity, primary language); (3) payer information (commercial, Medicare, Medicaid, self, or state-sponsored); (4) details of randomization, including the intervention assigned and results of risk screening, if applicable; (5) whether a patient was offered, accepted, and completed rapid HIV testing; (6) results from all rapid HIV tests; and for all patients with a reactive HIV test (7) confirmatory test results, CD4 counts and viral loads, whether they were successfully linked into care, and details of follow-up care and disease progression. Each site database will then be cleaned and have discrepancies resolved prior to replacing names and medical record numbers with unique patient identifiers.

Once each site database has been cleaned and stripped of patient identifiers, it will be transferred electronically to Denver Health Medical Center (the Data Coordinating Center) using a SFTP. All electronic databases will be stored in an electronic database (Microsoft SQL, Microsoft Corporation, Redmond, WA) that is password protected and stored on a firewall-protected and highly-secure server. Paper records will be stored in locked file cabinets at each individual site. All study-related materials (electronic files or paper records) will be accessible only by study investigators or authorized study personnel.

Procedures will be implemented to assure patient confidentiality in gathering and recording all study-related data, and close oversight of all collection, transfer, and data storage by the Principal Investigator, Project Managers, Data Coordinator, and site investigators. We will also invoke Data Use Agreements between the three study sites and Denver Health. In order to further minimize the risk of breach of

confidentiality, study data will not be placed or stored on a laptop computer or other portable storage unit (e.g., "jump drive"). All collected data will follow Denver Health and Hospital Authority security regulations for additional protection. Once complete, the database will be completely de-identified and all study-related documents with protected health information will be destroyed. No external data safety monitoring board will be used for this study.

B. Estimated Duration of the Study

It is estimated that this study will take up to 6 years (5 year study duration and 1 year follow-up). For patients enrolled during the course of this project, the estimated duration of their participation is the length of time spent in the ED at each participating ED. For those patients who test positive for HIV infection and participate in the follow-up portion of this project, the estimated duration of their participation will be 1 year following their initial HIV diagnosis (**Figure 2**).

Figure 2. Study timeline. Each site will begin enrollment following this proposed timeline. The months leading up to enrollment will involve capacity building and implementation planning. Enrollment will proceed until 3,500 HIV tests are performed at each site, for a total of 14,000 HIV tests. Dashed lines represent the likely scenario that enrollment will vary across institutions.

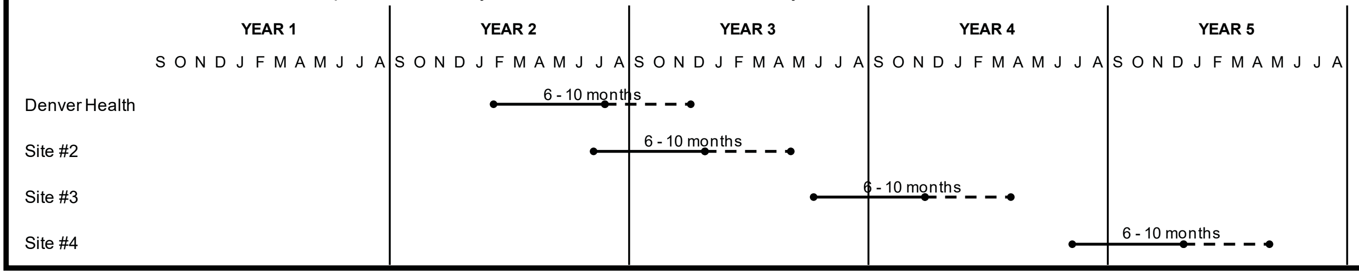

C. Number and Distribution of Subjects

As described above, enrollment will proceed until approximately 3,500 HIV tests are performed per site, for a total of 14,000 HIV tests. Therefore, it is estimated that up to 30,000 subjects per site will be included in this project, resulting in up to 120,000 total subjects included across all four sites. This will represent the largest ED-based HIV screening study to-date and the first to evaluate these three standard-or-care approaches. This will also allow each site to begin and end enrollment without committing to a specific time period (i.e., some sites may complete enrollment in 6 months, whereas other sites may require 10 months, depending on screen positive and HIV test completion rates) (**Figure 2**).

D. Examinations, Laboratory Tests, Procedures and Follow-Up Visits

All patients will receive standard medical care during their ED visits. Patients who test preliminarily positive for HIV infection will receive standard medical care for their HIV infection. This research in no way will change or alter standards of care for those who participate.

All patients who are offered and agree to rapid HIV testing will have blood drawn and sent to the institution's laboratory. Each patient's blood sample used for rapid HIV testing will be labeled with the patient's name, medical record and encounter number (as is the standard process at DHMC).

All rapid HIV testing will be performed using valid and Food and Drug Administration (FDA)-approved HIV testing methods. All of these tests are performed as a part of standard of care. All rapid HIV testing, will be performed by the hospital laboratory at DHMC with an approximate total turn-around time of 30 – 45 minutes, which includes preparation time, processing time, and reporting time. All HIV preliminary testing results will be provided to the patients during their ED visit.

This model also includes referring patients between 16 and 22 years of age or those who are pregnant to the Children's Hospital Colorado Immunodeficiency Program (CHIP) for primary medical care, and all patients ≥22 years old to the Infectious Diseases Clinic at DHMC for their initial medical evaluations. These evaluations include performing confirmatory testing and initial CD4 counts and viral loads. Patients who are residents of Denver County will then receive continuing medical care through either the Infectious Diseases Clinic (if the initial CD4 count is <200 cells/mm^3) or through the Denver Health HIV Primary Care Clinic (if the CD4 count is ≥200 cells/mm^3). Patients who reside outside Denver County will be referred to the Infectious Disease Group Practice at University of Colorado Hospital (UCH) for continuing medical care, and those patients who have other forms of medical insurance (e.g., private insurance) will be referred into their respective healthcare systems. All referral sites will provide individual level health education (ILHE) and prevention case management (PCM). In addition CDPHE will provide ILHE, PCM, substance abuse, mental health support, and partner counseling and referral services in parallel to those preventative care services provided by DHMC, UCH, or CHIP. These preventative care services are aimed at behavioral risk reduction, health education, promoting and reinforcing safer behaviors, and increasing the proportion of people infected with HIV and their partners who are linked into appropriate prevention care and treatment services.

Patients who test positive will be asked to provide written informed consent for the collection of additional information regarding HIV testing history and behavioral risk factors, as well as follow-up data pertaining to their medical treatment for HIV infection (see **"Longitudinal Data Collection Instrument" or "Limited Longitudinal Data Collection Instrument"** in the **Appendix)**. These follow-up data will be collected from the Infectious Diseases Clinic or the HIV Primary Care Clinic at DHMC, CHIP, or the Infectious Disease Group Practice at UCH. Physicians from each of these sites serve as co-investigators for this study.

For patients assigned to either targeted HIV screening arm, in which a risk assessment tool is utilized to target higher risk patients for screening, those identified as higher-risk and either receive a negative rapid HIV test result or decline HIV testing will be advised of the need for periodic retesting and will be offered or referred for prevention counseling.

E. Protected Health Information

Protected health information will be included on most documents used in this study. Such information will include each patient's names, medical record and encounter number, dates of birth, sex, race/ethnicity, date of visit, registration time, and discharge or hospitalization time, and the result of the rapid HIV test (if performed). In addition, for patients who test positive for HIV infection and agree to participate in the follow-up portion of this study, additional information will be collected, including laboratory results (i.e., CD4 count and viral load), additional behavioral risk factor information, and details of follow-up care, including scheduled and maintained medical care visits, unscheduled medical visits, information pertaining to a previous diagnosis of HIV infection, and, if clinically indicated, whether highly-active antiretroviral therapy (HAART) and treatment for opportunistic infections were initiated, the number of hospitalizations and follow-up visits in the 12 month follow-up period, the last documented CD4 count and the date, as well as mortality.

F. Risks

Subjects

The most significant risk to patients will be loss of confidentiality, which is considered minimal. All of the methods utilized in this study are currently accepted, recommended and utilized methods for HIV screening, locally or nationally. All medical centers in Colorado, including DHMC, are required by state law to report all diagnoses of HIV infection to the state health department. Patients who access testing services in routine health care settings are exposed to this same risk. Therefore, the risks involved in this study are no more than what the patient would experience in standard care. The Principal Investigator will assume full responsibility for the protection of all study-related documents, including those that contain protected health information.

Investigators/Institutions

None.

G. Benefits

The potential risks to subjects are outweighed by the possible benefit, which includes knowledge of HIV serostatus, and linkage-to-care for HIV positive patients. The benefit to society includes an increased number of individuals in the community who know their HIV status, and the possible reduction of forward transmission of this disease. The importance of the knowledge gained by conducting this study is that the results will substantially improve our understanding of how to perform effective and efficient rapid HIV screening in EDs and to predominantly underserved populations, and will inform similar practices in other high-risk care settings.

H. Limitations

Specific Aim 1

The primary difficulties with this aim relate to implementation of the HIV screening interventions in four high-volume EDs, the large number of included patients, and the consistent and accurate acquisition of their data. Our research team has extensive experience evaluating a wide range of HIV screening methods and acquiring large quantities of valid patient-level data. All members of the study team have experience overseeing and successfully completing large prospective ED-based HIV screening and multi-center studies. An additional potential difficulty relates to the heterogeneity of screening approaches used during this trial. Although heterogeneity may attenuate the effect of the interventions, this trial was specifically designed to evaluate effectiveness and to thus allow flexibility in how screening is performed in order to maximize external validity and generalizability.

Specific Aim 2

Methods used in this aim will rely on implementation of the three HIV screening methods described in Specific Aim 1. Potential difficulties will stem from performing manual time-motion data collection in four busy EDs and across a spectrum of days of the week and times of day. To maximize validity, we will use trained research assistants at each site and structured data collection procedures, similar to those previously used by our research team.[46] We will also randomly select data collection periods from all days of the week and times of day to obtain representative data. Also, although we will use actual costs associated with HIV screening, we realize that some costs may be artificial because of the research nature of the work; as such, we will perform sensitivity analyses to further characterize the costs and cost effectiveness of each screening approach. Finally, it is not our intention to perform cost modeling; instead, we plan to use an intermediate health outcome with the intent of extending the findings to quality-adjusted life years and specific societal scenario analyses in the near future. Development of robust and valid

decision and transition-state models will require focused resources that will be the objective of a future investigator-initiated grant.

Specific Aim 3

Valid and reliable acquisition of large quantities of data will be the principal difficulty with this aim. Given the large number of observations, it is not feasible or practical to collect data by hand. Instead, we will use electronic patient tracking systems from each ED to secure real-time data from all patients. We appreciate the quantity of data may be difficult to obtain, but a strength of our plan is that we have successfully used similar methodologies in the ED at Denver Health.[61] Dedicated staff members, including registration personnel, ED clerks, and nurses routinely enter real-time patient-specific data into each of the systems. All such staff members undergo formal training prior to being eligible to perform such data entry. A potential limitation of this process is that such data entry is not performed solely for research purposes, and thus may suffer from entry or coding biases. Given the large number of observations, we do not anticipate introduction of systematic bias. It is also possible that missing data will exist. If so, multiple imputation will be used as such statistical techniques are widely described and allow for an unbiased parameter estimates.[68-70]

I. Data Monitoring Plan

Data Safety Monitoring Plan

The study investigators will make every effort to keep each patient's data safe and confidential. The Project Coordinator and Principal Investigator will oversee all data collection. All electronic data will be transferred from the electronic patient tracking system in the ED into an electronic database (Microsoft SQL, Microsoft Corporation, Redmond, WA) using the protected network at DHMC. The database will be password protected and stored on a password-protected computer in the Project Coordinator's office in the administrative offices of the Department of Emergency Medicine at DHMC. Additional data will be collected from patients who test positive for HIV infection, as described above, and who consent for participation in the follow-up aspect of this project. These data will be recorded on closed-response data collection instruments, and stored in a locked file cabinet in the Project Coordinator's office. These data will be manually entered into the main electronic database. During all aspects of this project, all data will be kept in secure locations either in the office of the Principal Investigator, the office of the Project Coordinator, the office of the clinical social workers, or in the laboratory. The Principal Investigator will take full responsibility for the protection of all documents. The electronic database will be stored on a password-protected computer in a locked room and accessible only by study investigators for either data entry or data analysis. In order to further minimize the risk of breach of confidentiality, study data will not be put on a laptop computer or other portable storage unit (e.g., "jump drive"). All collected data will follow Denver Health and Hospital Authority security regulations for additional protection. Once complete, this database will be completely de-identified and all study-related documents with protected health information will be destroyed.

Because of the "pragmatic" nature of the clinical trial and because all study procedures are considered minimal risk (see above), we do not anticipate requiring a data safety and monitoring board (DSMB) for this study. According to the National Institute of Allergy and Infectious Diseases (NIAID) Standard Operating Procedures for human subjects' protection, "[i]f you are conducting a multisite clinical trial involving interventions that pose more than minimal risk to participants, you must have a…DSMB to monitor the trial".

J. Summarize Knowledge to be Gained

Results will help inform public health practices of how best to identify patients with undiagnosed HIV infection in EDs in the United States and provide important information related to improving the timeliness of diagnosis of HIV infection and linkage-to-care.

6. REFERENCES

1. HIV in the United States. 2011. (Accessed December 28, 2011, at http://www.cdc.gov/hiv/resources/factsheets/PDF/us.pdf.)
2. Prejean J, Song R, Hernandez A, et al. Estimated HIV Incidence in the United States, 2006–2009. PLoS ONE 2011;6:e17502.
3. Cohen MS, Chen YQ, McCauley M, et al. Prevention of HIV-1 infection with early antiretroviral therapy. N Engl J Med 2011;365:493-505.
4. Crepaz N, Hart TA, Marks G. Highly active antiretroviral therapy and sexual risk behavior: a meta-analytic review. Jama 2004;292:224-36.
5. Marks G, Crepaz N, Senterfitt JW, Janssen RS. Meta-Analysis of High-Risk Sexual Behavior in Persons Aware and Unaware They are Infected with HIV in the United States: Implications for HIV Prevention Programs. J Acquir Immune Defic Syndr 2005;39:446-53.
6. Branson BM, Handsfield HH, Lampe MA, et al. Revised recommendations for HIV testing of adults, adolescents, and pregnant women in health-care settings. MMWR Recomm Rep 2006;55:1-17; quiz CE1-4.
7. Bartlett JG, Branson BM, Fenton K, Hauschild BC, Miller V, Mayer KH. Opt-Out Testing for Human Immunodeficiency Virus in the United States: Progress and Challenges. Jama 2008;300:945-51.
8. Centers for Disease Control and Prevention. Results of the Expanded HIV Testing Initiative--25 jurisdictions, United States, 2007-2010. MMWR Morb Mortal Wkly Rep 2011;60:805-10.
9. High-Impact HIV Prevention: CDC's Approach to Reducing HIV Infection in the United States. 2011. (Accessed September 27, 2011, at http://www.cdc.gov/hiv/nhas/dhap/pdf/nhas_booklet.pdf.)
10. National HIV/AIDS Strategy for the United States. 2010. (Accessed September 27, 2011, at http://www.whitehouse.gov/sites/default/files/uploads/NHAS.pdf.)
11. Rothman RE, Lyons MS, Haukoos JS. Uncovering HIV Infection in the Emergency Department: A Broader Perspective. Academic Emergency Medicine 2007;14:653-7.
12. Pitts SR, Niska RW, Xu J, Burt CW. National Hospital Ambulatory Medical Care Survey: 2006 emergency department summary. Natl Health Stat Report 2008:1-38.
13. Pitts SR, Carrier ER, Rich EC, Kellermann AL. Where Americans get acute care: increasingly, it's not at their doctor's office. Health Aff (Millwood) 2010;29:1620-9.
14. Jenkins TC, Gardner EM, Thrun MW, Cohn DL, Burman WJ. Risk-based human immunodeficiency virus (HIV) testing fails to detect the majority of HIV-infected persons in medical care Settings. Sex Transm Dis 2006;33:329-33.
15. Centers for Disease Control and Prevention. Revised guidelines for HIV counseling, testing, and referral. MMWR Recomm Rep 2001;50:1-57; quiz CE1-19a1-CE6-a1.
16. Hsieh YH, Rothman RE, Newman-Toker DE, Kelen GD. National estimation of rates of HIV serology testing in US emergency departments 1993-2005: baseline prior to the 2006 Centers for Disease Control and Prevention recommendations. Aids 2008;22:2127-34.
17. Kelen GD, Hexter DA, Hansen KN, et al. Feasibility of an emergency department-based, risk-targeted voluntary HIV screening program. Ann Emerg Med 1996;27:687-92.
18. Kendrick SR, Kroc KA, Couture E, WEinstein RA. Comparison of Point-of-Care Rapid HIV Testing in Three Clinical Venues. Aids 2004;18:2208-10.
19. Lyss SB, Branson BM, Kroc KA, Couture EF, Newman DR, Weinstein RA. Detecting Unsuspected HIV Infection with a Rapid Whole-Blood HIV Test in an Urban Emergency Department. J Aquir Immune Defic Syndr 2007;44:435-42.
20. Lyons MS, Lindsell CJ, Ledyard HK, Frame PT, Trott AT. Emergency department HIV testing and counseling: an ongoing experience in a low-prevalence area. Ann Emerg Med 2005;46:22-8.
21. Brown J, Shesser R, Simon G, et al. Routine HIV Screening in the Emergency Department Using the New US Centers for Disease Control and Prevention Guidelines: Results from a High-Prevalence Area. J Acquir Immune Defic Syndr 2007;46:395-401.

22. Merchant RC, Seage GR, Mayer KH, Clark MA, DeGruttola VG, Becker BM. Emergency department patient acceptance of opt-in, universal, rapid HIV screening. Public Health Rep 2008;123 Suppl 3:27-40.
23. Haukoos JS, Hopkins E, Eliopoulos VT, et al. Development and implementation of a model to improve identification of patients infected with HIV using diagnostic rapid testing in the emergency department. Acad Emerg Med 2007;14:1149-57.
24. Calderon Y, Haughey-Barrios M, Bijur P, Nestor J, Bauman L. HIV Testing in the Emergency Department: An Alternative Way to Increase Rates of Testing When Counselors Are Not Available. In: 2005 SAEM Annual Meeting. New York City, New York; 2005:175.
25. Calderon Y, Haughey M, Bijur P, et al. A Randomized Controlled Trial Evaluating the Educational Effectiveness of a Rapid Human Immundoeficiency Virus Posttest Counseling Video. In: 2006 SAEM Annual Meeting. San Francisco, California; 2006:S22.
26. Mehta SD, Hall J, Lyss SB, Skolnik PR, Pealer LN, Kharasch S. Adult and pediatric emergency department sexually transmitted disease and HIV screening: programmatic overview and outcomes. Acad Emerg Med 2007;14:250-8.
27. Arbelaez C, Block B, Losina E, et al. Rapid HIV testing program implementation: lessons from the emergency department. Int J Emerg Med 2009;2:187-94.
28. Rothman RE, Hsieh YH, Harvey L, et al. 2009 US Emergency Department HIV Testing Practices. Ann Emerg Med 2011;58 Suppl 1:S3-S9 e4.
29. Haukoos JS, Hopkins E, Hull A, et al. HIV Testing in Emergency Departments in the United States: A National Survey. Ann Emerg Med 2011;58 Suppl 1:S10-S6 e8.
30. Lyons MS, Lindsell CJ, Haukoos JS, et al. Nomenclature and definitions for emergency department human immunodeficiency virus (HIV) testing: report from the 2007 conference of the National Emergency Department HIV Testing Consortium. Acad Emerg Med 2009;16:168-77.
31. Centers for Disease Control and Prevention. Additional recommendations to reduce sexual and drug abuse-related transmission of human T-lymphotropic virus type III/lymphadenopathy-associated virus. MMWR Morb Mortal Wkly Rep 1986;35:152-5.
32. Rothman KJ, Greenland S. Modern Epidemiology. 2nd ed. Philadelphia: Lippincott Williams & Wilkins; 1998.
33. Haukoos J. Impact of Non-Targeted HIV Screening in Emergency Departments and the Ongoing Need for Targeted Strategies. Arch Intern Med 2011:(In Press).
34. d'Almeida KW, Kierzek G, de Truchis P, et al. Modest Public Health Impact of Nontargeted Human Immunodeficiency Virus Screening in 29 Emergency Departments. Arch Intern Med 2011.
35. World Health Organization. Guidance on provider-initiated HIV testing and counselling in health facilities. Switzerland: World Health Organization; 2007 May 2007.
36. Chou R, Huffman L. Screening for human immunodeficiency virus: focused update of a 2005 systematic review for the U.S. Preventive Services Task Force. Prepared for the Agency for Healthcare Research and Quality by the Oregon Evidence-based Practice Center at the Oregon Health and Science University, Portland, Oregon, under Contract No. 290-02-0024, Task Order Number 1. Rockville, Maryland: Agency for Healthcare Research and Quality; 2007 April 2007.
37. Moyer VA. Screening for HIV: U.S. Preventive Services Task Force Recommendation Statement. Annals of Internal Medicine 2013;N/A:N/A-N/A.
38. Irvin CB, Flagel BT, Fox JM. The emergency department is not the ideal place for routine HIV testing. Ann Emerg Med 2007;49:722.
39. Haukoos JS. Rethinking How We Perform HIV Testing in the Emergency Department. Ann Emerg Med 2011;58 Suppl 1:S160-3.
40. Haukoos JS, Lyons MS. Idealized models or incremental program evaluation: translating emergency department HIV testing into practice. Acad Emerg Med 2009;16:1044-8.
41. Joelving F. Don't screen everybody for HIV in the ER. Reuters 2011 October 24, 2011.
42. Joelving F. Opt-out HIV tests find few new infections. Reuters 2010.
43. Smith M. IAC: 'Opt-out' HIV tests have modest success. Medpage Today 2010.
44. . (Accessed December 28, 2011, at http://denveredhiv.org/.)
45. Haukoos JS, Hopkins E, Conroy AA, et al. Routine opt-out rapid HIV screening and detection of HIV infection in emergency department patients. Jama 2010;304:284-92.

46. Conroy A, Hopkins E, Byyny R, et al. Cost effectiveness of routine opt-out rapid HIV screening in the emergency department: results from a prospective controlled clinical trial. Acad Emerg Med 2008;16:S147-S8.
47. Haukoos JS, Hopkins E, Bender B, et al. Use of kiosks and patient understanding of opt-out and opt-in consent for routine rapid HIV screening in the emergency department. Acad Emerg Med 2011:(In Press).
48. Haukoos JS, Lyons MS, Lindsell CJ, et al. Derivation and Validation of the Denver HIV Risk Score for Targeted HIV Screening. Am J Epidemiol:(In Press).
49. Haukoos JS, Hopkins E, Bender B, Al-Tayyib A, Thrun M, for the Denver Emergency Department HIV Testing Research Consortium. Enhanced targeted HIV screening using the Denver HIV Risk Score outperforms nontargeted screening in the emergency department. In: Conference on Retroviruses and Opportunistic Infections; 2012 March, 2012; Seattle, Washington; 2012.
50. Haukoos J, Hopkins E, Bucossi M, et al. Validation of the refined Denver HIV risk score using a national HIV testing cohort. (In Preparation).
51. Schwartz D, Lellouch J. Explanatory and pragmatic attitudes in therapeutical trials. J Chronic Dis 1967;20:637-48.
52. Zwarenstein M, Treweek S, Gagnier JJ, et al. Improving the reporting of pragmatic trials: an extension of the CONSORT statement. BMJ 2008;337:a2390.
53. Damschroder LJ, Aron DC, Keith RE, Kirsh SR, Alexander JA, Lowery JC. Fostering implementation of health services research findings into practice: a consolidated framework for advancing implementation science. Implement Sci 2009;4:50.
54. Overview of Wage Data by Area and Occupation. 2011. (Accessed December 30, 2011, at http://www.bls.gov/bls/blswage.htm.)
55. Bernstein SL, Verghese V, Leung W, Lunney AT, Perez I. Development and validation of a new index to measure emergency department crowding. Acad Emerg Med 2003;10:938-42.
56. Centers for Disease Control and Prevention. Public Health Service guidelines for counseling and antibody testing to prevent HIV infection and AIDS. MMWR Morb Mortal Wkly Rep 1987;36:509-15.
57. Centers for Disease Control and Prevention. Recommendations for HIV testing services for inpatients and outpatients in acute-care hospital settings. Center for Disease Control and Prevention. MMWR Recomm Rep 1993;42:1-6.
58. White DA, Scribner AN, Vahidnia F, et al. HIV Screening in an Urban Emergency Department: Comparison of Screening Using an Opt-In Versus an Opt-Out Approach. Ann Emerg Med 2011;58 Suppl 1:S89-95.
59. Hsieh YH, Jung JJ, Shahan JB, et al. Outcomes and Cost Analysis of 3 Operational Models for Rapid HIV Testing Services in an Academic Inner-City Emergency Department. Ann Emerg Med 2011;58 Suppl 1:S133-9.
60. Delaney KP, Heffelfinger JD, Wesolowski LG, et al. Performance of an alternative laboratory-based algorithm for HIV diagnosis in a high-risk population. J Clin Virol 2011;52 Suppl 1:S5-S10.
61. Haukoos JS, Hopkins E, Byyny RL, et al. Design and implementation of a controlled clinical trial to evaluate the effectiveness and efficiency of routine opt-out rapid human immunodeficiency virus screening in the emergency department. Acad Emerg Med 2009;16:800-8.
62. Weiss SJ, Derlet R, Arndahl J, et al. Estimating the degree of emergency department overcrowding in academic medical centers: results of the National ED Overcrowding Study (NEDOCS). Acad Emerg Med 2004;11:38-50.
63. Weiss SJ, Ernst AA, Nick TG. Comparison of the National Emergency Department Overcrowding Scale and the Emergency Department Work Index for quantifying emergency department crowding. Acad Emerg Med 2006;13:513-8.
64. Grembowski D. The Practice of Health Program Evaluation. Thousand Oaks: Sage Publications, Inc.; 2001.
65. Drummond MF, O'Brien B, Stoddart GL, Torrance GW. Methods for the Economic Evaluation of Health Care Programmes. New York: Oxford University Press, Inc.; 2000.
66. Campbell MK, Torgerson DJ. Bootstrapping: estimating confidence intervals for cost-effectiveness ratios. Qjm 1999;92:177-82.
67. Gold MR, Siegel JE, Russell LB, Weinstein MC. Cost-Effectiveness in Health and Medicine. New York: Oxford University Press, Inc.; 1996.

68. Little RJA, Rubin DB. Statistical Analysis with Missing Data. 2nd ed. New Jersey: John Wiley & Sons, Inc.; 2002.
69. Haukoos JS, Newgard CD. Advanced statistics: missing data in clinical research--part 1: an introduction and conceptual framework. Acad Emerg Med 2007;14:662-8.
70. Newgard CD, Haukoos JS. Advanced statistics: missing data in clinical research--part 2: multiple imputation. Acad Emerg Med 2007;14:669-78.
