# Supplementary material for: Comparison of HIV Screening Strategies in the Emergency Department: A Randomized Clinical Trial
Source: JAMA Netw Open. 2021 Jul 26;4(7):e2117763. doi: 10.1001/jamanetworkopen.2021.17763 (PMC8314142; doi:10.1001/jamanetworkopen.2021.17763)
Supplement: Supplement 2. — eTable 1. Denver HIV Risk Score eTable 2. Traditional Targeted HIV Screening Questions eTable 3. Baseline Characteristics of Ineligible, Eligible but Not Randomized, and Randomized Patients eTable 4. Baseline Characteristics of Patients Randomized to Enhanced Targeted and Traditional Targeted Arms, Stratified by Risk eTable 5. Test Offer and Completion Among Patients Randomized to Each Study Arm, All Sites eTable 6. Outcomes During 12 Months Following HIV Diagnosis, Among All New HIV Diagnosed Patients by Study Arms, All Sites eTable 7. Outcomes During 12 Months Following HIV Diagnosis, Among All Patients Who Underwent Diagnostic Testing, All Sites eFigure 1. Enrollment From Denver Health Medical Center eFigure 2. Enrollment From the University of Cincinnati Medical Center eFigure 3. Enrollment From Johns Hopkins Hospital, Baltimore, Maryland eFigure 4. Enrollment From Highland Hospital, Oakland, California [file jamanetwopen-e2117763-s002.pdf]

Supplemental Online Content

Haukoos JS, Lyons MS, Rothman RE, et al; HIV TESTED Trial Investigators. Comparison of HIV screening strategies in the emergency department: a randomized clinical trial. *JAMA Netw Open.* 2021;4(7):e2117763. doi:10.1001/jamanetworkopen.2021.17763

eTable 1. Denver HIV Risk Score

eTable 2. Traditional Targeted HIV Screening Questions

eTable 3. Baseline Characteristics of Ineligible, Eligible but Not Randomized, and Randomized Patients

eTable 4. Baseline Characteristics of Patients Randomized to Enhanced Targeted and Traditional Targeted Arms, Stratified by Risk

eTable 5. Test Offer and Completion Among Patients Randomized to Each Study Arm, All Sites

eTable 6. Outcomes During 12 Months Following HIV Diagnosis, Among All New HIV Diagnosed Patients by Study Arms, All Sites

eTable 7. Outcomes During 12 Months Following HIV Diagnosis, Among All Patients Who Underwent Diagnostic Testing, All Sites

eFigure 1. Enrollment From Denver Health Medical Center

eFigure 2. Enrollment From the University of Cincinnati Medical Center

eFigure 3. Enrollment From Johns Hopkins Hospital, Baltimore, Maryland

eFigure 4. Enrollment From Highland Hospital, Oakland, California

This supplemental material has been provided by the authors to give readers additional information about their work.

© 2021 Haukoos JS et al. *JAMA Network Open.*

eTable 1. Denver HIV Risk Score.

Variable	Score
Age	
22-25 or 55-60 years	+4
26-32 or 47-54 years	+10
33-46 years	+12
Gender	
Male	+21
Race/Ethnicity	
Black	+9
Hispanic	+3
Sexual Practices	
Sex with a male	+22
Other Risks	
Injection drug use	+9
Past HIV test	-4

*Reference groups that score zero are:
Age, <22 or >60 years; Gender, female; Race/Ethnicity, white or "other", defined as American or Alaskan Native, Native Hawaiian, or non-Hawaiian Pacific Islander.
Dashes represent exclusion of the variable from the refined score. The refined score ranges from -4 to +73 with risk groups stratified as <20 (very low risk), 20 – 29 (low risk), 30 – 39 (moderate risk), 40 – 49 (high risk, and ≥50 (very high risk).

© 2021 Haukoos JS et al. *JAMA Network Open.*

eTable 2. Traditional targeted HIV screening questions. An affirmative response to any of the questions was considered increased risk for HIV. Adapted from the Centers for Disease Control and Prevention.*

Questions
**1.** Have you ever had vaginal or anal sex without a condom with someone that you think might be infected with HIV?
**2.** Have you ever had vaginal or anal sex without a condom with more than one sex partner?
**3.** Have you ever been diagnosed or treated for an STD, hepatitis, or tuberculosis?
**4.** Have you ever injected drugs and shared equipment with others?
**5.** Have you ever been told you have an infection related to a weak immune system?

Abbreviations: STD = sexually transmitted disease.
*Centers for Disease Control and Prevention. Revised guidelines for HIV counseling, testing, and referral. MMWR Recomm Rep 2001;50:1-57.

© 2021 Haukoos JS et al. *JAMA Network Open.*

eTable 3. Baseline characteristics of ineligible, eligible but not randomized, and randomized patients. Demographics are reported at the patient-level (N = 109,634) and emergency department characteristics at the visit level (N = 157,684).

	**Ineligible**		**Eligible but Not Randomized**		**Randomized**	
	**n**	**(%)**	**n**	**(%)**	**n**	**(%)**
**Patient-level characteristics**	**N = 35,143**		**N = 15,405**		**N = 59,086**	
Age, years (median, IQR)	32	(15 – 50)	38	(26 – 54)	40	(28 – 58)
Sex						
Female	15,993	(46)	7,073	(46)	30,302	(51)
Male	19,143	(54)	8,302	(54)	28,782	(49)
Unknown / Missing	7	(0)	30	(0)	2	(0)
Race/Ethnicity						
Asian, non-Hispanic	1,811	(5)	740	(5)	1,622	(3)
Black, non-Hispanic	10,363	(29)	5,235	(34)	22,827	(39)
Hispanic (all races)	11,294	(32)	2,796	(18)	12,616	(21)
White, non-Hispanic	9,831	(28)	5,333	(35)	19,478	(33)
Other	879	(3)	416	(3)	1,273	(2)
Unknown / Missing	965	(3)	885	(6)	1,270	(2)
Payer						
Commercial	4,658	(13)	2,477	(16)	12,928	(22)
Medicaid	19,946	(57)	6,135	(40)	26,146	(44)
Medicare	3,577	(10)	1,882	(12)	7,245	(12)
Self-Pay	4,617	(13)	2,458	(16)	7,467	(13)
Other	1,202	(3)	1,396	(9)	2,040	(3)
Unknown / Missing	1,143	(3)	1,057	(7)	3,260	(6)
**Visit-level characteristics**	**N = 54,668**		**N = 26,455**		**N = 76,561**	
Mode of Arrival						
Ambulatory	39,939	(73)	14,411	(54)	57,698	(75)
EMS	12,320	(23)	8,610	(33)	15,880	(21)
Unknown / Missing	2,409	(4)	3,434	(13)	2,983	(4)
Acuity						
ESI Level 1	630	(1)	1,362	(5)	697	(1)
ESI Level 2	9,133	(17)	6,762	(26)	9,542	(12)
ESI Level 3	18,996	(35)	11,274	(43)	47,912	(63)
ESI Level 4	20,469	(37)	5,307	(20)	16,723	(22)
ESI Level 5	5,312	(10)	929	(4)	1,609	(2)
Unknown / Missing	128	(0)	821	(3)	78	(0)

Abbreviations: IQR = interquartile range; EMS = emergency medical services; ESI = Emergency Severity Index.

© 2021 Haukoos JS et al. *JAMA Network Open.*

eTable 4. Baseline characteristics of patients randomized to enhanced targeted and traditional targeted arms, stratified by risk. Demographics are reported at the patient-level and emergency department characteristics at the visit level.

	Enhanced Targeted				Traditional Targeted			
	High Risk		Low Risk		High Risk		Low Risk	
	n	(%)	n	(%)	n	(%)	n	(%)
**Patient-level characteristics**	N = 12,588		N = 10,028		N = 6,699		N = 15,991	
Age, years (median, IQR)	39	(30 – 49)	44	(24 – 61)	36	(27 – 50)	42	(29 – 55)
Sex								
Female	6,015	(48)	5,489	(55)	3,263	(49)	8,385	(52)
Male	6,537	(52)	4,539	(45)	3,436	(51)	7,604	(48)
Unknown / Missing	0	(0)	0	(0)	0	(0)	2	(0)
Race/Ethnicity								
Asian, non-Hispanic	215	(2)	387	(4)	89	(1)	503	(3)
Black, non-Hispanic	6,278	(50)	2,522	(25)	3,190	(48)	5,877	(37)
Hispanic (all races)	2,734	(22)	2,129	(21)	1,001	(15)	3,787	(24)
White, non-Hispanic	2,921	(23)	4,513	(45)	2,238	(33)	5,117	(32)
Other	223	(2)	,243	(2)	106	(2)	374	(2)
Unknown / Missing	217	(2)	234	(2)	75	(1)	333	(2)
Payer								
Commercial	2,343	(19)	2,477	(25)	1,360	(20)	3,620	(23)
Medicaid	6,526	(52)	3,911	(39)	3,508	(52)	6,886	(43)
Medicare	1,145	(9)	1,716	(17)	654	(10)	2,194	(14)
Self-Pay	1,608	(13)	1,037	(10)	674	(10)	1,946	(12)
Other	364	(2)	309	(3)	178	(3)	545	(3)
Unknown / Missing	602	(5)	578	(6)	325	(5)	800	(5)
**Visit-level characteristics**	N = 13,883		N = 11,570		N = 7,099		N = 18,540	
Mode of Arrival								

© 2021 Haukoos JS et al. *JAMA Network Open.*

	Enhanced Targeted				Traditional Targeted			
	**High Risk**		**Low Risk**		**High Risk**		**Low Risk**	
	**n**	**(%)**	**n**	**(%)**	**n**	**(%)**	**n**	**(%)**
Ambulatory	10,637	(77)	8,585	(74)	5,434	(77)	13,926	(75)
EMS	2,635	(19)	2,628	(23)	1,347	(19)	3,951	(21)
Unknown / Missing	611	(4)	359	(3)	318	(4)	663	(4)
Acuity								
ESI Level 1	100	(1)	135	(1)	57	(1)	173	(1)
ESI Level 2	1,502	(6)	1,644	(14)	780	(11)	2,462	(13)
ESI Level 3	8,785	(63)	7,127	(62)	4,458	(63)	11,558	(62)
ESI Level 4	3,193	(23)	2,396	(21)	1,654	(23)	3,940	(21)
ESI Level 5	299	(2)	247	(2)	145	(2)	382	(2)
Unknown / Missing	4	(0)	21	(0)	5	(0)	25	(0)

Abbreviations: IQR = interquartile range; EMS = emergency medical services; ESI = Emergency Severity Index.

© 2021 Haukoos JS et al. *JAMA Network Open.*

eTable 5. Test offer and completion among patients randomized to each study arm, all sites.

	Nontargeted (N = 25,469)		Enhanced Targeted (N = 25,453)		Traditional Targeted (N = 25,639)	
	**n**	**(%)**	**n**	**(%)**	**n**	**(%)**
HIV Screening						
“High Risk”*	NA		13,883	(54.5)	7,099	(27.7)
“Low Risk”†	NA		11,570	(45.5)	18,540	(72.3)
Opt-Out HIV Screening‡	25,160	(98.8)	13,702	(98.7)	7,073	(99.6)
Did Not Decline HIV Testing	9,313	(37.0)	6,010	(43.9)	4,164	(58.9)
Completed HIV Testing	6,744	(72.4)	4,488	(74.7)	3,173	(76.2)
Diagnostic HIV Testing						
Completed Diagnostic Testing	423	(1.7)	666	(2.6)	723	(2.8)
Among those who were not offered§	14	(4.5)	422	(3.6)	608	(3.3)
Among those who opted-out¶	409	(2.6)	244	(3.2)	115	(4.0)

Abbreviations: NA = not applicable.

*Defined as a Denver HIV Risk Score ≥30 for those in the enhanced targeted group or as any affirmative response to one of five behavioral risk questions in the traditional targeted group, as adapted from the Centers for Disease Control and Prevention and the World Health Organization.

†Defined as a Denver HIV Risk Score <30 for those in the enhanced targeted group or no affirmative response to one of the five behavioral risk questions in the traditional targeted group.

‡309 (1.2%) patients in the nontargeted group, 181 (1.3%) in the enhanced targeted group, and 26 (0.4%) in the traditional targeted group were not notified that HIV testing would be performed.

§Patients who were not notified that testing would be performed in the nontargeted group (n = 309) or those identified as “low risk” or “high risk” and not notified that testing would be performed in either targeted arm (enhanced targeted, n = 11,751; traditional targeted, n = 18,566).

¶Patients who opted-out of testing in the nontargeted group (n = 15,538) or those identified as “high risk” in either targeted arm but opted- out of testing (enhanced targeted, n = 7,692; traditional targeted, n = 2,909).

© 2021 Haukoos JS et al. *JAMA Network Open.*

eTable 6. Outcomes during 12 months following HIV diagnosis, among all new HIV diagnosed patients by study arms, all sites.

	Nontargeted (N = 10)		Enhanced Targeted (N = 7)		Traditional Targeted (N = 7)	
	**n**	**(%)**	**n**	**(%)**	**n**	**(%)**
Longitudinal Outcomes						
Linked to care*	8	(80)	7	(100)	7	(100)
Initiated ART	4	(40)	6	(86)	5	(71)
AIDS Diagnosis	1	(10)	1	(14)	2	(29)
Alive at 12 months	9	(90)	5	(71)	6	(88)
Unscheduled visits†	1	(1 – 3)	1.5	(0 – 5)	0	(0 – 1)
Hospitalizations†	1	(0 – 5)	1	(0 – 3)	1	(1 – 6)
CD4						
Initial CD4 (cells/μL)†	224	(203 – 417)	365	(305 – 414)	359	(20 – 640)
Last CD4 (cells/μL)†	583	(343 – 867)	523	(410 – 560)	177	(140 – 558)
Viral Load						
Initial viral load (copies/mL)†	34,788	(20,627 – 80,671)	15,900	(4,833 – 27,415)	106,000	(786 – 519,233)
Last viral load (copies/mL)†	6,540	(0 – 30,400)	27	(0 – 734)	0	(0 – 0)

Abbreviations: ART = antiretroviral therapy; AIDS = acquired immunodeficiency syndrome; IQR = interquartile range.
*Defined as completing at least one HIV medical care visit.
†Reported as median and IQR.

© 2021 Haukoos JS et al. *JAMA Network Open.*

eTable 7. Outcomes during 12 months following HIV diagnosis, among all patients who underwent diagnostic testing, all sites.

	Included Patients (N = 1,812)				Not Included Patients (N = 2,656)			
	New Diagnoses (n = 7)		Repeat Diagnoses (n = 7)		New Diagnoses (n = 17)		Repeat Diagnoses (n = 13)	
Longitudinal Outcomes	N	(%)	N	(%)	N	(%)	N	(%)
Linked to care*	5	(71.4)	5	(71.4)	15	(88.2)	12	(92.3)
Initiated ART	3	(42.9)	3	(42.9)	13	(76.5)	6	(46.2)
AIDS Diagnosis	4	(57.1)	3	(42.9)	2	(11.8)	2	(15.4)
Alive at 12 months†	3	(42.9)	7	(58.3)	17	(100.0)	11	(84.6)
Unscheduled visits‡	3	(0 – 3)	0	(0 – 0.5)	1	(0 – 2)	2	(0 – 3)
Hospitalizations†	0	(0 – 0)	19.5	(11.5 – 24.5)	0	(0 – 2)	1	(1 – 5)
CD4								
Initial CD4 (cells/μL)‡	35	(26 – 111)	303	(162 – 408)	424	(378 – 609)	66	(21 – 237)
Last CD4 (cells/μL)‡	201	(164 – 224)	120	(51 – 336)	850	(622 – 962)	334	(145 - 442)
Viral Load								
Initial viral load (copies/mL)‡	109,653	(57,200 – 180,000)	46,196	(17,800 – 69,280)	394,331	(37,290 – 1,975,000)	25,780	(219 – 1,730,409)
Last viral load (copies/mL)‡	20	(0 – 445)	61	(39 – 664)	0	(0 – 0)	0	(0 – 800)

Abbreviations: ART = antiretroviral therapy; AIDS = acquired immunodeficiency syndrome; IQR = interquartile range.
*Defined as completing at least one HIV medical care visit.
†2 (8%) of the 24 new diagnoses and 1 (5%) of the 20 repeat diagnoses were lost to follow-up at 12 months.
‡Reported as median and IQR.

© 2021 Haukoos JS et al. *JAMA Network Open.*

eFigure 1. Enrollment from Denver Health Medical Center, Denver, Colorado. Parentheses represent percentages.

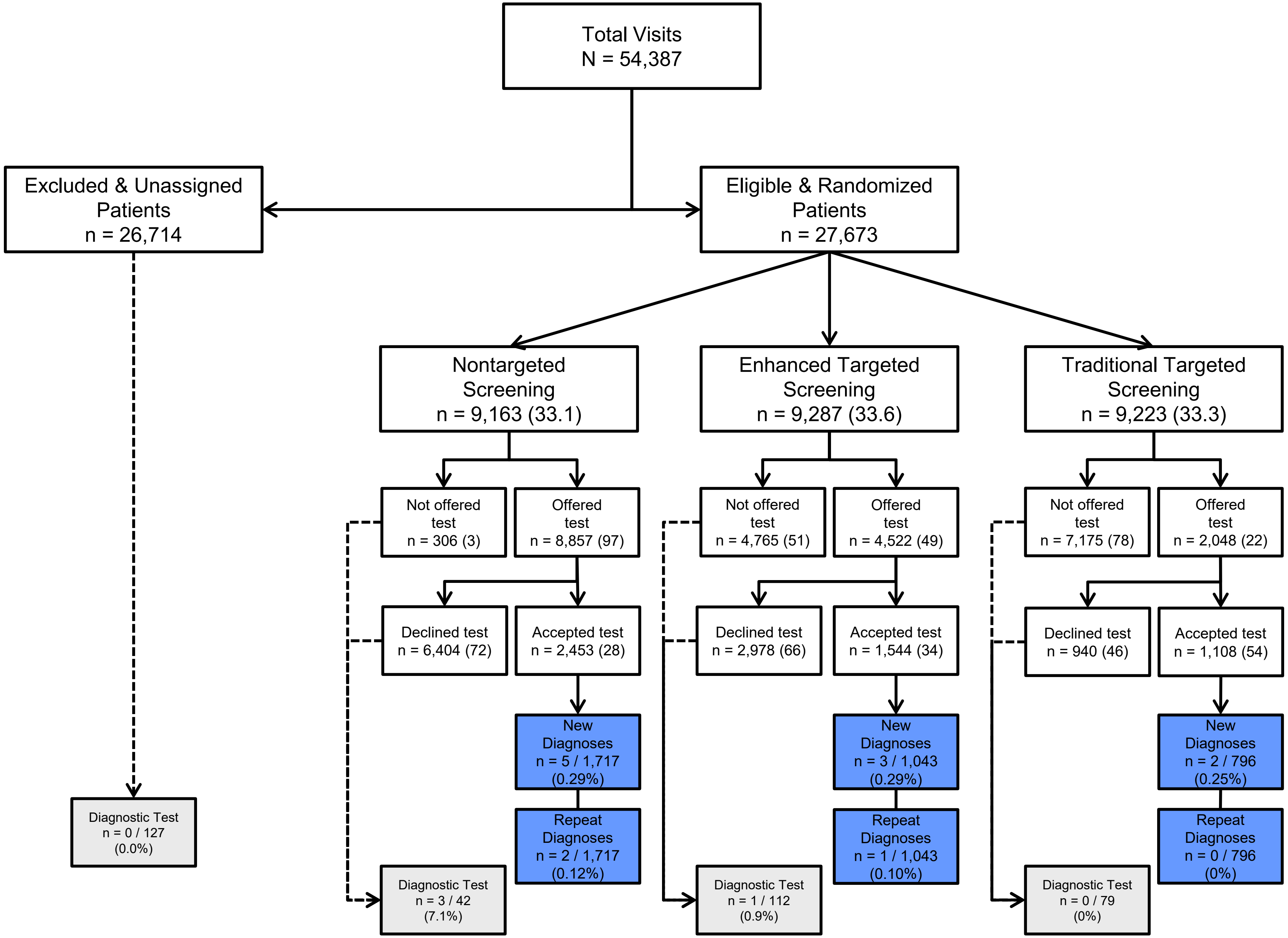

© 2021 Haukoos JS et al. *JAMA Network Open.*

eFigure 2. Enrollment from the University of Cincinnati Medical Center, Cincinnati, Ohio. Parentheses represent percentages.

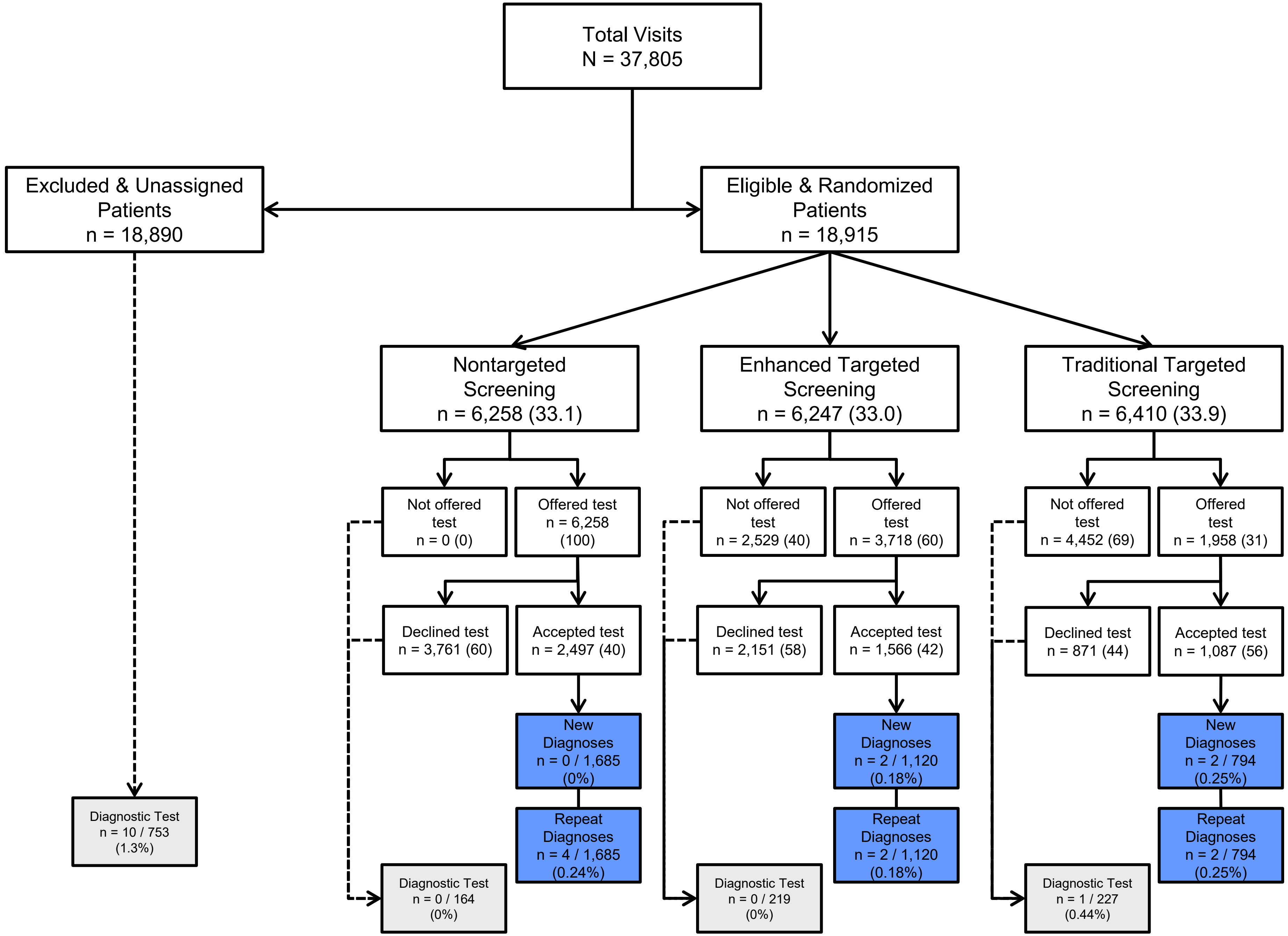

© 2021 Haukoos JS et al. *JAMA Network Open.*

eFigure 3. Enrollment from Johns Hopkins Hospital, Baltimore, Maryland. Parentheses represent percentages.

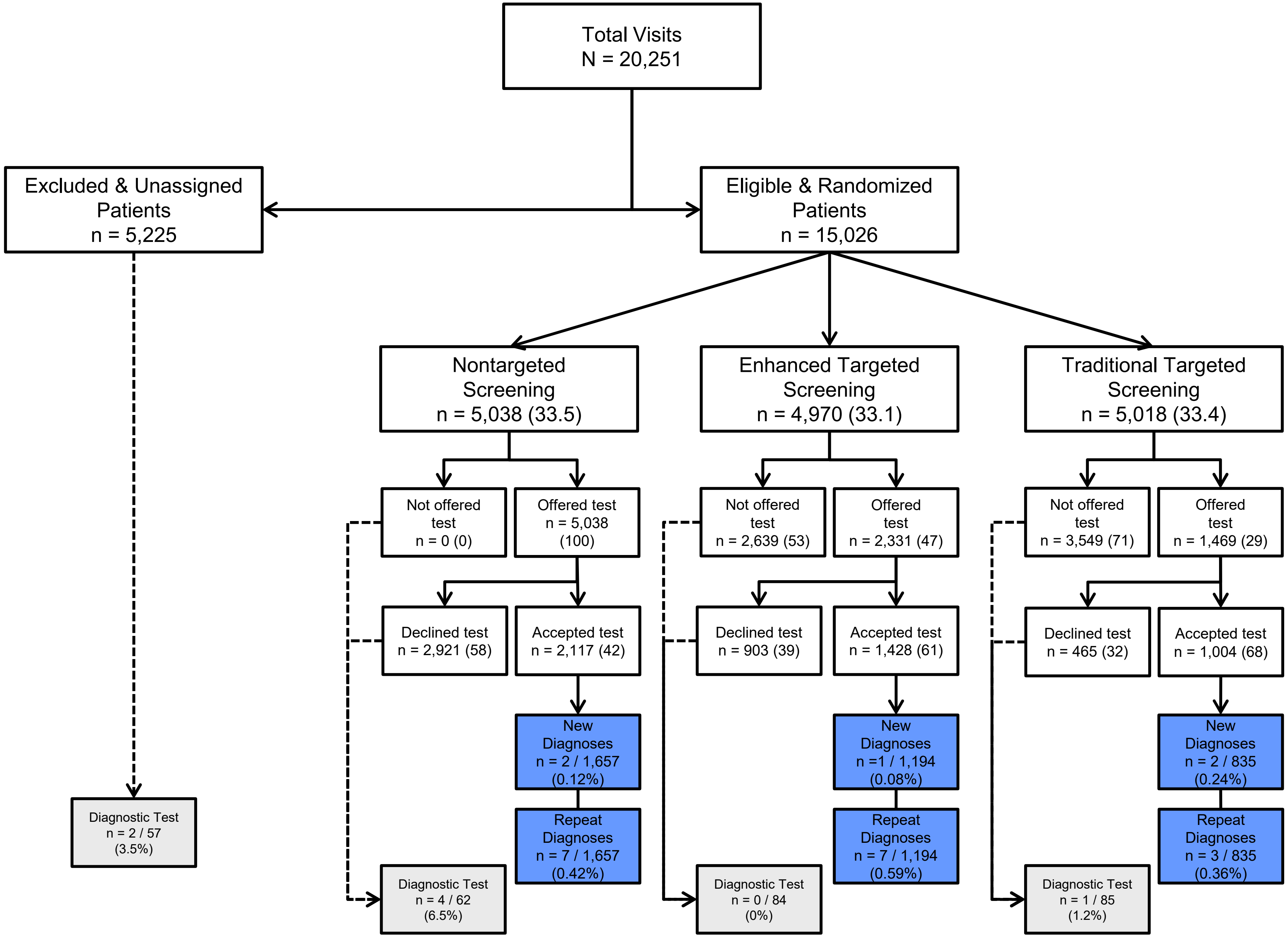

© 2021 Haukoos JS et al. *JAMA Network Open.*

eFigure 4. Enrollment from Highland Hospital, Oakland, California. Parentheses represent percentages.

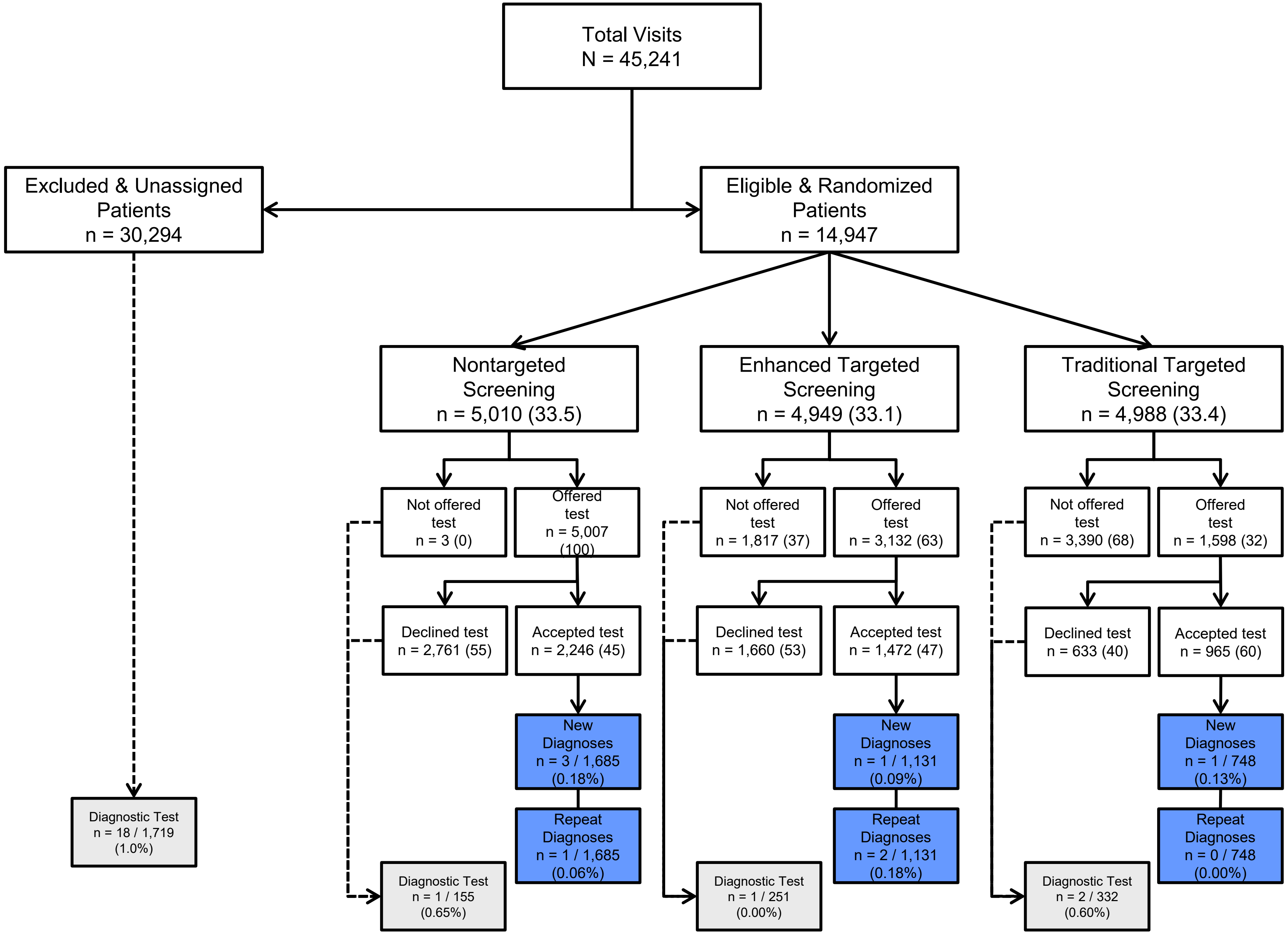

© 2021 Haukoos JS et al. *JAMA Network Open.*
