# Supplementary material for: Comparison of HIV Screening Strategies in the Emergency Department: A Randomized Clinical Trial
Source: JAMA Netw Open. 2021 Jul 26;4(7):e2117763. doi: 10.1001/jamanetworkopen.2021.17763 (PMC8314142; doi:10.1001/jamanetworkopen.2021.17763)
Supplement: Supplement 3. — Nonauthor Collaborators. The HIV TESTED (Testing using Enhanced Screening Techniques in Emergency Departments) Trial Investigators [file jamanetwopen-e2117763-s003.pdf]

Supplemental Online Content: Nonauthor Collaborators

*Indicates required information. Only first name, last name, and suffix will appear in PubMed.

*Group Name(s): The HIV TESTED (Testing using Enhanced Screening Techniques in Emergency Departments) Trial Investigators							
*First Name and Middle Initial(s)	*Last Name	*Suffix (eg, Jr, III)	Academic Degrees	Institution	Location (city, state/province, country)	Role or Contribution, eg, chair, principal investigator	Group (if more than 1 Group listed in the byline) and/or Subgroup (eg, Steering Committee)
Bryan	Austin		MHA	Johns Hopkins University	Baltimore, MD	Informational Tech	
Jacob	Cohen		MD	Johns Hopkins University	Baltimore, MD	Research Assistant	
Jackie	Easley		MSN	Johns Hopkins University	Baltimore, MD	Nurse Education	
Somiya	Haider		MD	Johns Hopkins University	Baltimore, MD	Research Coordinator	
Peter	Hill		MD	Johns Hopkins University	Baltimore, MD	Medical Director	
Mauren	Henley		MSN	Johns Hopkins University	Baltimore, MD	Nurse Education	
Gabor	Kelen		MD	Johns Hopkins University	Baltimore, MD	Departmental Chair	
Maggie	Leathers		MS	Johns Hopkins University	Baltimore, MD	Research Coordinator	
Barbara	Maliszewski		MSN	Johns Hopkins University	Baltimore, MD	Nurse Education	
Paula	Neira		JD MSN	Johns Hopkins University	Baltimore, MD	Nurse Education	
Scott	Newton		DNP MHA	Johns Hopkins University	Baltimore, MD	Nursing Director	
Lucas	Spaeth		MS PA-C	Johns Hopkins University	Baltimore, MD	Research Assistant	
Tina	Tolson		MSN	Johns Hopkins University	Baltimore, MD	Nursing Director	
Valentina	Viertel		MPH	Johns Hopkins University	Baltimore, MD	Research Coordinator	
Maddie	Whalen		MSN MPH	Johns Hopkins University	Baltimore, MD	Nurse Education	
Lindsay	Bohanske		MPH	University of Cincinnati Medical Center	Cincinnati, OH	Research Coordinator	
Catherine	Cronin		MT(ASCP)	University of Cincinnati Medical Center	Cincinnati, OH	Content Expert	
Frank	Fernandez		MD	University of Cincinnati Medical Center	Cincinnati, OH	Informational Tech	
Carl	Fichtenbaum		MD	University of Cincinnati Medical Center	Cincinnati, OH	Content Expert	
Cortney	Gaffney		BSN	University of Cincinnati Medical Center	Cincinnati, OH	Content Expert	
Cathy	Hamilton		MPH	University of Cincinnati Medical Center	Cincinnati, OH	Informational Tech	
Kim	Hart		MA	University of Cincinnati Medical Center	Cincinnati, OH	Informational Tech	
Dave	Hoskins		BS	University of Cincinnati Medical Center	Cincinnati, OH	Informational Tech	
Sharon	Kohrs		BSN	University of Cincinnati Medical Center	Cincinnati, OH	Content Expert	
Christopher	Lindsell		PhD	University of Cincinnati Medical Center	Cincinnati, OH	Content Expert	
Chris	Miller		MD MS	University of Cincinnati Medical Center	Cincinnati, OH	Content Expert	
Brittany	Punches		PhD	University of Cincinnati Medical Center	Cincinnati, OH	Content Expert	
Geri	Rowan		BSN	University of Cincinnati Medical Center	Cincinnati, OH	Informational Tech	
Kim	Vance		MSN RN	University of Cincinnati Medical Center	Cincinnati, OH	Content Expert	
Teresa	Williams		MHA MT(ASCP)	University of Cincinnati Medical Center	Cincinnati, OH	Content Expert	
Steve	Cantrill		MD	Denver Health Medical Center	Denver, CO	Informational Tech	
Angelica	Chavez		BSN	Denver Health Medical Center	Denver, CO	Clinical Champion	
Ann	Comeau		MSW	Denver Health Medical Center	Denver, CO	Social Work Manager	
Tracy	Daugherty		BSN	Denver Health Medical Center	Denver, CO	Clinical Champion	
Alex	Delgado		BSW	Denver Health Medical Center	Denver, CO	Linkage-to-Care	
Pamela	Doyle		BSN	Denver Health Medical Center	Denver, CO	Nurse Education	

Supplemental Online Content: Nonauthor Collaborators

*Indicates required information. Only first name, last name, and suffix will appear in PubMed.

*First Name and Middle Initial(s)	*Last Name	*Suffix (eg, Jr, III)	Academic Degrees	Institution	Location (city, state/province, country)	Role or Contribution, eg, chair, principal investigator	Group (if more than 1 Group listed in the byline) and/or Subgroup (eg, Steering Committee)
Roberto	Esquivel			Denver Health Medical Center	Denver, CO	Linkage-to-Care	
Maren	Fassino			Denver Health Medical Center	Denver, CO	Informational Tech	
Kelly	Finnegan		MD	Denver Health Medical Center	Denver, CO	Research Assistant	
Greg	Fliney		MD	Denver Health Medical Center	Denver, CO	Research Assistant	
Theresa	Freudig		MSN	Denver Health Medical Center	Denver, CO	Nurse Education	
Michael	Fuhriman			Denver Health Medical Center	Denver, CO	Linkage-to-Care	
Mary	Gillman		MBA MHA	Denver Health Medical Center	Denver, CO	Laboratory Support	
Stacy	Kahl-Geiger		LCSW	Denver Health Medical Center	Denver, CO	Linkage-to-Care	
Korina	Keating		MSW	Denver Health Medical Center	Denver, CO	Linkage-to-Care	
Jason	Koerner		BS	Denver Health Medical Center	Denver, CO	Research Assistant	
Karen	Laber		BSN	Denver Health Medical Center	Denver, CO	Clinical Champion	
Meghan	Lang		MSW	Denver Health Medical Center	Denver, CO	Linkage-to-Care	
Carolynn	Lyle		PA-C MPH	Denver Health Medical Center	Denver, CO	Research Assistant	
Kim	Makela		MSW	Denver Health Medical Center	Denver, CO	Linkage-to-Care	
Mary	Maltby		BSN	Denver Health Medical Center	Denver, CO	Clinical Champion	
David	Medina		RN	Denver Health Medical Center	Denver, CO	Nurse Director	
Tanya	Nielsen		LCSW	Denver Health Medical Center	Denver, CO	Linkage-to-Care	
Alice	Ortiz			Denver Health Medical Center	Denver, CO	ED Administration	
Angela	Paolucci		RN	Denver Health Medical Center	Denver, CO	Clinical Champion	
Don	Pate			Denver Health Medical Center	Denver, CO	Clinical Champion	
Michael	Pippins		BS	Denver Health Medical Center	Denver, CO	Informational Tech	
Steven	Roy		RN	Denver Health Medical Center	Denver, CO	Clinical Champion	
John	Searcey		RN	Denver Health Medical Center	Denver, CO	Clinical Champion	
Jerry	Solot		MD	Denver Health Medical Center	Denver, CO	Medical Director	
Kelly	Stermer		BSN	Denver Health Medical Center	Denver, CO	Nurse Education	
Brian	Stuart		ADN	Denver Health Medical Center	Denver, CO	Clinical Champion	
Melissa	Swanson		BSN	Denver Health Medical Center	Denver, CO	Clinical Champion	
Lauren	Timkovich		MSW	Denver Health Medical Center	Denver, CO	Linkage-to-Care	
Zenia	Toure		MSW	Denver Health Medical Center	Denver, CO	Linkage-to-Care	
Lisa	Vogel		BSN MBA	Denver Health Medical Center	Denver, CO	Nurse Director	
Sommer	Walker		BSN	Denver Health Medical Center	Denver, CO	Nurse Director	
Diane	Weed		MA MT(ASCP)	Denver Health Medical Center	Denver, CO	Laboratory Support	
Julia	Weise		MSW	Denver Health Medical Center	Denver, CO	Linkage-to-Care	
Kerri	Wenke		BSN	Denver Health Medical Center	Denver, CO	Nurse Champion	
Courtney	Wham		MS	Denver Health Medical Center	Denver, CO	Research Assistant	
Michael	Wilson		MD	Denver Health Medical Center	Denver, CO	Laboratory Director	
Jarrod	Wright		BSN	Denver Health Medical Center	Denver, CO	Nurse Director	
Melody	Zwakenberg		BSN ANP-BC	Denver Health Medical Center	Denver, CO	Clinical Champion	

Supplemental Online Content: Nonauthor Collaborators

*Indicates required information. Only first name, last name, and suffix will appear in PubMed.

***First Name and Middle Initial(s)**	***Last Name**	***Suffix (eg, Jr, III)**	Academic Degrees	Institution	Location (city, state/province, country)	Role or Contribution, eg, chair, principal investigator	Group (if more than 1 Group listed in the byline) and/or Subgroup (eg, Steering Committee)
Harrison	Alter		MD	Highland Hospital, Alameda Health System	Oakland, CA	Content Expert	
Erik	Anderson		MD	Highland Hospital, Alameda Health System	Oakland, CA	Content Expert	
Brendan	Campbell		BA	Highland Hospital, Alameda Health System	Oakland, CA	Research Assistant	
Grace	Chang		BA	Highland Hospital, Alameda Health System	Oakland, CA	Research Assistant	
Connie	Chao		CLS	Highland Hospital, Alameda Health System	Oakland, CA	Laboratory Support	
Katie	Ellis		BA	Highland Hospital, Alameda Health System	Oakland, CA	Research Assistant	
Sarah	Graffman		RN	Highland Hospital, Alameda Health System	Oakland, CA	Nurse Champion	
Brynn	Kron		BA	Highland Hospital, Alameda Health System	Oakland, CA	Research Assistant	
Rebecca	Lucas		RN	Highland Hospital, Alameda Health System	Oakland, CA	Nurse Education	
Kaitlin	McCarthy		RN	Highland Hospital, Alameda Health System	Oakland, CA	Linkage-to-Care	
Valeria	Ng		MD PhD	Highland Hospital, Alameda Health System	Oakland, CA	Laboratory Director	
Christine	O'Dell		RN	Highland Hospital, Alameda Health System	Oakland, CA	Nurse Champion	
Jim	Peck			Highland Hospital, Alameda Health System	Oakland, CA	Informational Tech	
Mae	Petti		BA	Highland Hospital, Alameda Health System	Oakland, CA	Research Assistant	
Feuy	Saechao		CLS	Highland Hospital, Alameda Health System	Oakland, CA	Laboratory Support	
Barry	Simon		MD	Highland Hospital, Alameda Health System	Oakland, CA	Departmental Chair	
Tonya	Tyree		RN	Highland Hospital, Alameda Health System	Oakland, CA	Linkage-to-Care	
