# Supplementary material for: Comparison of HIV Screening Strategies in the Emergency Department: A Randomized Clinical Trial
Source: JAMA Netw Open. 2021 Jul 26;4(7):e2117763. doi: 10.1001/jamanetworkopen.2021.17763 (PMC8314142; doi:10.1001/jamanetworkopen.2021.17763)
Supplement: Supplement 4. — Data Sharing Statement [file jamanetwopen-e2117763-s004.pdf]

Haukoos JS, Lyons MS, Rothman RE, et al; HIV TESTED Trial Investigators. Comparison of HIV screening strategies in the emergency department: a randomized clinical trial. *JAMA Netw Open.* 2021;4(7):e2117763. doi:10.1001/jamanetworkopen.2021.17763

Data Sharing Statement

Data

Data available: Yes
Data types: Deidentified participant data, Data dictionary
How to access data: jason.haukoos@dhha.org
When available: With publication

Supporting Documents

Document types: None

Additional Information

Who can access the data: Researchers who proposed use of the data has been approved.
Types of analyses: For any approved purpose.
Mechanisms of data availability: After approval of a proposal with signed data use agreement.
Any additional restrictions: None.
